# Japanese Classification of Esophageal Cancer, 11th Edition: part I

**DOI:** 10.1007/s10388-016-0551-7

**Published:** 2016-11-10

**Authors:** Japan Esophageal Society

**Affiliations:** Hirose-Building 4F, Taihei 2-3-13, Sumida-ku, Tokyo, 130-0012 Japan

*President*

Hisahiro Matsubara: Chiba University

*Former President*

Nobutoshi Ando: Tokyo Dental University

*English Edition Committee, Chairman*

Hisahiro Matsubara: Chiba University

*English Edition Committee Members*

Kenji Nemoto: Yamagata University
Naohisa Yahagi: Keio University
Soji Ozawa: Tokai University
Yoshiaki Kajiyama: Juntendo University
Tatsuyuki Kawano: Tokyo Medical and Dental University
Tomio Arai: Tokyo Metropolitan Geriatric Hospital and Institute of Gerontology
Yuji Tachimori: National Cancer Center Hospital
Shoji Natsugoe: Kagoshima University
Kumiko Momma: Tokyo Metropolitan Cancer and Infectious Diseases Center Komagome Hospital
Yasuyuki Seto: Tokyo University
Yuichiro Doki: Osaka University

*English Edition Supervisor*

Hiromasa Fujita: Fukuoka Wajiro Hospital

*Editorial Assistants*

Yasunori Akutsu: Chiba University

*Japanese Edition Committee, Chairman*

Hisahiro Matsubara: Chiba University

*Japanese Edition Committee Members*

Kenji Nemoto: Yamagata University
Naohisa Yahagi: Keio University
Soji Ozawa: Tokai University
Yoshiaki Kajiyama: Juntendo University
Tatsuyuki Kawano: Tokyo Medical and Dental University
Tomio Arai: Tokyo Metropolitan Geriatric Hospital and Institute of Gerontology
Yuji Tachimori: National Cancer Center Hospital
Shoji Natsugoe: Kagoshima University
Kumiko Momma: Tokyo Metropolitan Cancer and Infectious Diseases Center Komagome Hospital
Yasuyuki Seto: Tokyo University
Yuichiro Doki: Osaka University

*Pathological Research Committee, Chairman*

Tomio Arai: Tokyo Metropolitan Geriatric Hospital and Institute of Gerontology

*Pathological Research Committee Members*

Yasuo Ohkura: Kyorin University
Shingo Ishiguro: PCL Japan
Hiroshi Kawachi: The Cancer Institute Hospital of Japanese Foundation for Cancer Research
Kaiyo Takubo: Tokyo Metropolitan Institute of Gerontology
Masamitsu Unakami: Watari Hospital
Takashi Yao: Juntendo University
Suguru Yonezawa: Kagoshima University
Tetsuo Nemoto: Toho University

*Endoscopy Research Committee, Chairman*

Tuneo Oyama: Saku Central Hospital

*Endoscopy Research Committee Members*

Kumiko Momma: Tokyo Metropolitan Cancer and Infectious Diseases Center Komagome Hospital
Tai Omori: Kawasaki Municipal Ida Hospital
Tatsuyuki Kawano: Tokyo Medical and Dental University
Hideo Shimada: Tokai University Oiso Hospital
Manabu Takeuchi: Nagaoka Red Cross Hospital
Ken Haruma: Kawasaki Medical School
Ryu Ishihara: Osaka Medical Center for Cancer and Cardiovascular Diseases
Akio Yanagisawa: Kyoto Prefectural University of Medicine
Ryoji Kushima: Shiga University of Medical Science

**Contents**

Preface

General principles of this edition

Abbreviations

Part I General rules

1. Purpose, object, and methods of descriptions
  1.1. Purpose
  1.2. Object
  1.3. Methods of descriptions
    1.3.1. Principles of descriptions and abbreviations
2. Clinical aspects
  2.1. Description of primary tumor
    2.1.1. Number of primary tumors, size and circumferential location
    2.1.2. Tumor location
    2.1.3. Macroscopic tumor type
    2.1.4. Depth of tumor invasion (T)
  2.2. Metastatic lesions from esophageal cancerMetastatic lesions from esophageal cancer
    2.2.1. Lymph node metastasis
    2.2.2. Distant organ metastasis (M)
  2.3. Stage
  2.4. Multiple primary cancers
3. Surgical aspects
  3.1. Handling of the resected specimen
  3.2. Description of surgical findings and macroscopic findings of primary tumor
    3.2.1. Tumor size
    3.2.2. Distance from surgical margin to the tumor
    3.2.3. Macroscopic tumor type
    3.2.4. Surgical margin
    3.2.5. RM: Radial margin
  3.3. Intramural metastasis and multiple cancers of the esophagus
    3.3.1. IM: Intramural metastasis
    3.3.2. Multiple cancers of the esophagus
  3.4. Lymph nodes
    3.4.1. Preparation of resected lymph nodes for pathological examination
    3.4.2. Grading of lymph node metastasis (N)
    3.4.3. Lymph node dissection (D)
  3.5. Distant organ metastasis (M)
  3.6. Residual tumor (R)
  3.7. Curativity (Cur)
4. Pathological findings
  4.1. Handling of the surgically resected specimens
  4.2. Description of pathological findings
    4.2.1. Histological classification
    4.2.2. Depth of tumor invasion (pT)
    4.2.3. Infiltrative growth pattern (INF)
    4.2.4. Vascular invasion (ly/v)
    4.2.5. Intramural metastasis (pIM)
    4.2.6. Distance from surgical margin
    4.2.7. Multiple primary cancers
    4.2.8. Others
    4.2.9. Pathological criteria for the effects of radiation and/or chemotherapy
  4.3. Lymph node metastasis (pN)
  4.4. Distant organ metastasis (pM)
  4.5. Residual tumor (pR)
  4.6. Curativity (pCur)
5. Endoscopic treatment
  5.1. Handling of specimens resected endoscopically
  5.2. Description of macroscopic findings and endoscopic findings
    5.2.1 Number of tumors and number of resected specimens
    5.2.2 Size of resected specimen and size of tumor lesion (for each lesion)
    5.2.3 Tumor types
    5.2.4 Macroscopic findings
    5.2.5. Clinical assessment of residual tumor
  5.3. Preparation for pathological examination
  5.4. Description of pathological findings
    5.4.1. Pathological diagnosis
    5.4.2. Depth of tumor invasion (pT)
    5.4.3. Resection margin
    5.4.4. Infiltrative growth pattern (INF)
    5.4.5. Vascular invasion (ly/v)
    5.4.6. Report of pathological findings
  5.5. Residual tumor (pR)
  5.6. Curativity (pCur)
6. Barrett esophagus and adenocarcinoma in Barrett esophagus
  6.1. Definition and description methods for Barrett mucosa, Barrett esophagus and adenocarcinoma in Barrett esophagus
    6.1.1. Definition of the esophagogastric junction (EGJ)
    6.1.2. Barrett mucosa
    6.1.3. Barrett esophagus
    6.1.4. Adenocarcinoma in Barrett esophagus
  6.2. Tumor location
  6.3. Description of tumors
    6.3.1. Primary tumor
    6.3.2. Intramural metastasis (IM)
    6.3.3 Lymph node metastasis (N)
    6.3.4. Distant organ metastasis (M)
  6.4. Stage
7. Treatment
  7.1. Endoscopic treatment
    7.1.1. Endoscopic resection: ER
    7.1.2. Other endoscopic treatment
  7.2. Surgical treatments
    7.2.1. Resection and reconstruction procedures
    7.2.2. Conservative/palliative procedure
  7.3. Stenting
    7.3.1. Esophageal stents
    7.3.2. Tracheobronchial stents
    7.3.3. Aortic stents
  7.4. Common issues for radiotherapy and chemotherapy
    7.4.1. Disease status
    7.4.2. Aim of treatment
    7.4.3. Reasons for definitive radiotherapy
  7.5. Radiotherapy (RT)
    7.5.1. Clinical target volume (CTV)
    7.5.2. Methods of radiotherapy
    7.5.3. External beam radiotherapy
    7.5.4. Intraluminal irradiation
    7.5.5. Completion of treatment
    7.5.6. Reasons for treatment cessation
  7.6. Chemotherapy (CT)
    7.6.1. Agents
    7.6.2. Administration routes
    7.6.3. Administration procedures
    7.6.4. Administration doses
    7.6.5. Administration schedules
    7.6.6. Duration of administration
    7.6.7. Total administration dose
    7.6.8. Reasons for treatment cessation
    7.6.9. Adverse events
  7.7. Multi-modality treatment
    7.7.1. Combination of endoscopic treatment and surgery, radiotherapy, chemoradiotherapy or chemotherapy
    7.7.2 Chemoradiotherapy (CRT)
  7.8. Hyperthermia (HT)
  7.9. Immunotherapy (IT)
8. Results of treatment
  8.1. Total number of patients
  8.2. Multiple primary cancers
  8.3. Main treatment and adjuvant therapy
  8.4. Total number of patients treated, and number and ratio of patients treated with each procedure
    8.4.1. Patients operated
    8.4.2. Patients with endoscopic treatment
    8.4.3. Patients with chemotherapy and/or radiotherapy
  8.5. Operative mortality
  8.6. Hospital mortality
  8.7. Long-term outcome
    8.7.1. Alive or dead
    8.7.2. Recurrence
  8.8. Long-term outcomes and prognosis, especially survival rate
    8.8.1. Analysis of survival rates
    8.8.2. Period and rate of esophageal preservation
  8.9. Terminology related to survival period
    8.9.1. Survival time
    8.9.2. Overall survival (OS)
    8.9.3. Median survival time (MST)
    8.9.4. Survival rate
    8.9.5. Progression-free survival (PFS), time to progression (TTP)
    8.9.6. Relapse-free survival, recurrence-free survival (RFS)
    8.9.7. Disease-free survival (DFS)
    8.9.8. Time to treatment failure (TTF)
    8.9.9. Response duration
    8.9.10. Complete response duration

**Preface to the 11th Edition**

Eight years after the publication of the 10th edition in 2007, the 11th edition of the Japanese Classification of Esophageal Cancer has now been published. During this period, supplements to the 10th edition involving the revision of “disease typing” and terminology were published in 2008; in addition, following the adoption of criteria for the diagnosis of lesions located at the gastroesophageal junction that was made in cooperation with the Japanese Gastric Cancer Association, a 7-page leaflet was attached to this Classification in September 2013. The present revision was aimed at ensuring consistency with other general rules for surgical and pathological studies on cancer as far as possible, reflecting the latest advances in the diagnosis and treatment of esophageal cancer in Japan and providing a set of rules that are easier to use and that facilitate improvements in treatment outcomes. During this revision, we attempted to secure consistency with the UICC’s TNM classification as far as possible. However, this attempt was skipped for the N classification, since the current edition (7th) of the TNM classification does not reflect the nationwide registry data of the Japan Esophageal Society and because the rules for studies on supraclavicular lymph nodes are completely different between our classification and the N classification. This is a significant issue that will need to be addressed in the next revision.

Using nationwide registry data, the effects of regional lymph node excision were reviewed from the viewpoints of lymph node metastasis and the survival rate. As a result, the lymph node groupings were modified (T4 was subdivided into two subtypes, similar to the TNM classification). Following recent advances and the spread of endoscopic treatment, findings from endoscopic treatment have now been incorporated into the description methods, and the exclusion of cancer from intraepithelial neoplasms has been clarified. This revised edition has been prepared as a result of numerous discussions among committee members. Although there are still some questions to be discussed, we wish to take this opportunity to thank the considerable efforts made by the individual committee members.

October, 2015

**General principles of this edition**

1. Following the spread of endoscopic treatment, findings from endoscopic treatment (e) have been added to the methods used to describe findings.
2. The criteria for the diagnosis of lesions located at the gastroesophageal junction, which have been jointly adopted by the Japanese Gastric Cancer Association, have been added to the main text.
3. Regarding the depth of tumor invasion, the subgroup T1b- has been added to the subgroup T1b, similar to that for T1a, and the subgroup T4 has been further subdivided into T4a and T4b so as to be consistent with the UICC’s TNM classification.
4. Regarding lymph nodes, No. 112ao has been divided into the esophageal side and the dorsal side. Furthermore, to secure consistency with the general rules for surgical and pathological studies on gastric cancer, No. 3 has been divided into No. 3a and No. 3b.
5. Regarding lymph node grouping, modifications have been made to Ut (Group 3 only), Mt/Lt (Groups 1, 2, 3), and Ae (Groups 2, 3). In accordance with the revision of the criteria for the diagnosis of lesions located at the gastroesophageal junction, the same lymph node classification as that used for Ae will now be applied to cancer of the gastroesophageal junction.
6. Regarding the stage of cancer, T1aN1 is now classified as Stage II, as is the case with T1bN1. T4a up to N3 is now classified as Stage III. T4b, beginning with N0, is now classified as Stage IVa.
7. Regarding the extent of residual cancer, classification into R1 based on macroscopic findings is now avoided, consistent with the general rules for surgical and pathological studies on colorectal cancer.
8. Regarding histopathological findings, it has now been made clear that carcinoma in situ is not to be included among neoplasms within the squamous epithelium. The extent of differentiation of both squamous cell carcinoma and adenocarcinoma is now described as “well differentiated” or a similar expression, omitting any description of type. Endocrine cell neoplasm is now called neuroendocrine tumor, consistent with the WHO classification. Also, concerning extralymph node metastasis, the expression “tumor nodule” has been adopted, consistent with the general rules for surgical and pathological studies on colorectal cancer. Vascular invasion in specimens collected during endoscopic treatment is now rated as (−) or (+), consistent with the method used for gastric cancer.
9. The TNM classification adopted for the revised classification has been switched to the Japanese translation of the TNM classification, 7th edition.
10. Regarding the number of lymph node metastases, the conventional rule for the correction of grouping according to the number of metastases was too complex and was not used frequently. This rule has been deleted from the revised edition.
11. The endoscope pictures have been replaced with clearer ones.
12. Regarding the extents and borders of the lymph nodes, not only schematic figures, but also actual CT images have been provided to simplify understanding, accompanied by the presentation of features that will also be useful for radiotherapy.

**Abbreviations**
AD: Adventitia
Ae: Abdominal esophagus
AI: Invasion to the adjacent structures
APC: Argon plasma coagulation
B: Tracheal bifurcation
c: Clinical findings
Ce: Cervical esophagus
CR: Complete response
CRT: Chemoradiotherapy
CT: Chemotherapy
CTV: Clinical target volume
Cur: Curativity
D: Lymph node dissection
DFS: Disease-free survival
DM: Distal margin
DMM: Deep muscularis mucosae
E: Esophagus
EG: Tumor located in the esophageal side
EGJ: Esophagogastric junction
EMR: Endoscopic mucosal resection
EP: Epithelium [p. 41, 74]
ER: Endoscopic resection
ESD: Endoscopic submucosal dissection
EVG: Elastica van Gieson staining
f: Final findings
G: Stomach
GE: Tumor located in the gastric side
GIST: Gastrointestinal stromal tumor
H: Esophageal hiatus
HM: Horizontal margin
HT: Hyperthermia
IM: Intramural metastasis
INF: Infiltrative growth pattern
IR/SD: Incomplete response/stable disease
IT: Immunotherapy
LN: Lymph node
LPM: Lamina propria mucosae
LSBE: Long segment Barrett esophagus
Laser: Laser therapy
Lt: Lower thoracic esophagus
Ly: Lymphatic invasion
ly/v: Lymphatic invasion or venous invasion
M: Distant organ metastasis
MCT: Microwave coagulation therapy
MFH: Malignant fibrous histiocytoma
MM: Muscularis mucosae
MP: Muscularis propria
MST: Median survival time
Mt: Middle thoracic esophagus
N: Lymph node metastasis
O: Esophageal orifice
OS: Overall survival
p: Pathological findings
PD: Progressive disease
PDT: Photodynamic therapy
PFS: Progression-free survival
Ph: Pharynx
PM: Proximal margin
PR: Partial response
R: Residual tumor
RECIST: Response Evaluation Criteria in Solid Tumors
RFS: Relapse/recurrence-free survival
RM: Radial margin
RT: Radiotherapy
s: Surgical findings
S: Superior margin of the sternum
SCE: Specialized columnar epithelium
SCJ: Squamocolumnar junction
SD: Stable disease
SM: Submucosa
SMM: Superficial muscularis mucosae
SSBE: Short segment Barrett esophagus
T: Depth of tumor invasion
Te: Thoracic esophagus
Tis: Carcinoma in situ
TT: Thermotherapy
TTF: Time to treatment failure
TTP: Time to progression
Ut: Upper thoracic esophagus
v: Venous invasion
VB: Victoria blue staining
VM: Vertical margin [p. 39]
X: Cannot be assessed [p. 2]

**Terminology of the lymph nodes**
R: Right
L: Left
sm: Submandibular
spf: Superficial
ac: Accessory
tr: Tracheal
up: Upper
mid: Middle
rec: Recurrent nerve
tb: Tracheobronchial
pre: Pretracheal
ao: Paraaortic
pul: Pulmonary ligament

Number of the lymph nodes

a: 1–3, b: 4–7, c: ≧8

**Part I**

**General rules**

**1. Purpose, object, and methods of descriptions**

1.1. Purpose

“The Guidelines for the Clinical and Pathologic Studies on Carcinoma of the Esophagus” was originally published in 1969 by the Japanese Society for Esophageal Diseases. Since then, the Society has changed its name in 2003 to become the Japan Esophageal Society, and has published the “Japanese Classification of Esophageal Cancer” in Japanese with some revisions to keep up to date with treatment results and to provide a standard nomenclature. To promote the international use of the Guidelines and the Classification, the Society is publishing this handbook in English entitled “The Japanese Classification of Esophageal Cancer”.

1.2. Object

The term esophageal cancer in the *Japanese Classification* refers to cancer originating in the esophagus, and cancer metastatic to the esophagus is excluded. All primary malignant tumors in the esophagus should be described according to the *Japanese Classification*.

1.3. Methods of descriptions

*1.3.1. Principles of descriptions and abbreviations*

Findings are recorded using upper-case letters T (depth of tumor invasion), N (lymph node metastasis) and M (distant organ metastasis). The extent of each finding is expressed by Arabic numerals following each upper-case letter. “X” is used in unknown cases. Five categories of findings, namely Clinical, Endoscopic treatment, Surgical, Pathological, and Final findings, are identified using the lower case “c”, “e”, “s”, “p”, and “f”, respectively, before each upper-case letter. The “f” of Final findings can be omitted (Tables 1-1, 1-2). Checklists for descriptions of the Japanese Classification of Esophageal Cancer are shown in the following tables (Tables 1-3, 1-4).

**Table 1-1:** Principles of description

Clinical findings (c)	Endoscopic treatment findings (e)	Surgical findings (s)	Pathological findings (p)	Final findings (f)
Physical examination Diagnostic imaging Xray, Endoscopy (NBI magnification, Iodine staining, EUS etc.), CT, MRI, PET etc. Biopsy and Cytology Biochemical and Biological examinations Others (genetic studies etc.)	Operative findings Macroscopic examination of the resected specimens	Operative findings Intraoperative diagnostic imaging Frozen sections Macroscopic examination of the resected specimens	Pathological examination of materials obtained by surgical or endoscopic resection	Comprehensive findings based on clinical, surgical and pathological findings Note

Note: In cases in which any findings are modified by combined treatment, findings should be recorded as the estimated most advanced condition throughout the treatment

Tracheal invasion was observed from clinical findings; cT4, cN2, cM0, cStage IVa

As the tumor responded to chemoradiotherapy, surgery was performed; CRT-sT3, sN2, sM0, sStage III

Although the tumor completely responded to chemoradiotherapy on pathological findings, metastasis to Group 3 lymph nodes was observed; CRT-pT0 (T3), pN3, sM0, pStage III

Final findings; (f) T4, (f) N3, (f) M0, (f) Stage IVa

**Table 1-2:** Description methods

	Clinical findings	Endoscopic treatment findings	Surgical findings	Pathological findings	Final findings
Depth of tumor invasion	cT	eT	sT	pT	(f) T
Lymph node metastasis	cN	–	sN	pN	(f) N
Distant organ metastasis	cM	–	sM	pM	(f) M
Intramural metastasis	cIM	eIM	sIM	pIM	(f) IM
Stage	cStage	eStage	sStage	pStage	(f) Stage
Proximal margin	–	–	sPM	pPM	(f) PM
Distal margin	–	–	sDM	pDM	(f) DM
Radial margin	–	–	sRM	pRM	(f) RM
Horizontal margin (EMR/ESD)	–	eHM	sHM	pHM	(f) HM
Vertical margin (EMR/ESD)	–	eVM	sVM	pVM	(f) VM
	–				
Residual tumor	–	eR	sR	pR	(f) R
Curativity	–	eCur	sCur	pCur	(f) Cur

Findings modified by treatment methods other than surgery are abbreviated as follows:* RT* radiotherapy,* CT* chemotherapy,* CRT* chemoradiotherapy,* EMR* endoscopic mucosal resection, *ESD* endoscopic submucosal dissection, *laser* laser therapy,* PDT* photodynamic therapy

**Table 1-3:** Checklist for descriptions of the Japanese Classification of Esophageal Cancer (surgically treated cases)

Tumor location: Ce, Ut, Mt, Lt, Ae
Size: maximum length (mm) and orthogonally oriented maximum width (mm)
Macroscopic tumor type:
Type 0, Type 1, Type 2, Type 3, Type 4, Type 5, Combined type, Others
Subclassification for superficial cancers: 0-I(0-Ip, 0-Is), 0-II(0-IIa, 0-IIb, 0-IIc), 0-III
Histological type: squamous cell carcinoma, basaloid (-squamous) carcinoma, carcinosarcoma, adenocarcinoma, Barrett's adenocarcinoma, adenosquamous carcinoma, mucoepidermoid carcinoma, adenoid cystic carcinoma, neuroendocrine cell tumor, undifferentiated carcinoma, other carcinomas, non-epithelial malignant tumors, GIST, malignant melanoma
Depth of tumor invasion: pTX, pT0, pT1a (EP, LPM, MM), pT1b (SM1, SM2, SM3), pT2, pT3, pT4
Pattern of infiltration: INFa, INFb, INFc
Lymphatic invasion: ly0, ly1, ly2, ly3
Venous invasion: v0, v1, v2, v3
Intramural metastasis: pIMX, pIM0, pIM1
Involvement of resection margins
Proximal margin: pPMX, pPM0, pPM1
Distal margin: pDMX, pDM0, pDM1
Radial margin: pRMX, pRM0, pRM1
Multiple primary cancers: absent, present (number)
Lymph node metastasis: pNX, pN0, pN1, pN2, pN3, pN4
Number of positive nodes (No. of lymph node stations with positive nodes)
Distant metastasis: M0, M1
Residual tumor: pRX, pR0, pR1, pR2
Histological curativity: pCurA, pCurB, pCurC
Distant organ metastasis: MX, M0, M1; Intramural gastric metastasis: pIM1-St
Therapeutic efficacy: No data, Grade 0, Grade 1a, Grade 1b, Grade 2, Grade 3
Curativity: Cur A, Cur B, Cur C

**Table 1-4:** Checklist for descriptions of the Japanese Classification of Esophageal Cancer (endoscopically treated cases)

Macroscopic findings
Tumor location: Ce, Ut, Mt, Lt, Ae
Macroscopic tumor type: Type 0-I, Type 0-IIa, Type 0-IIb, Type 0-IIc, Type 0-III, combined type, others
Size of specimen: length (mm), width (mm)
Size of tumor: length (mm), width (mm)
Resection: en bloc resection, piecemeal resection
Piecemeal resection: number of specimens
Horizontal margin: HMX, HM0, HM1
Vertical margin: VMX, VM0, VM1
Multiple lesions: present, absent
Histological findings
Histological type: squamous cell carcinoma, basaloid (-squamous) carcinoma, carcinosarcoma, adenocarcinoma, Barrett's adenocarcinoma, adenosquamous carcinoma, mucoepidermoid carcinoma, adenoid cystic carcinoma, neuroendocrine cell tumor, undifferentiated carcinoma, other carcinomas, non-epithelial malignant tumors, GIST, malignant melanoma
Depth of tumor invasion: pTX, pT0, pT1a (EP, LPM, MM), T1b (SM1, SM2, SM3)
Pattern of infiltration: INFa, INFb, INFc
Lymphatic invasion: ly (−), ly (+)
Venous invasion: v (−), v (+)
Size of tumor: length (mm) × width (mm)
Horizontal margin: pHMX, pHM0, pHM1
Vertical margin: pVMX, pVM0, pVM1
Residual tumor: pRX, pR0, pR1, pR2
Curativity: pCur A, pCur B, pCur C

The order of clinicopathological description is:

Tumor location (in addition to describing the distance from the incisor), circumferential extent, tumor length, macroscopic tumor type, histological type (when identified), depth of tumor invasion, lymph node metastasis, distant organ metastasis and stage.

e.g.: Mt (31–36 cm), 1/2 of circumference and on anterior wall, 5 cm, Type 2, moderately differentiated squamous cell carcinoma, pT3, pN2, sM0, fStage III.

The order of pathological description is:

Tumor location, tumor length, macroscopic tumor type, histological type, depth of tumor invasion, pattern of infiltration, lymphatic invasion, venous invasion, intramural metastasis, involvement of the resection margins (proximal margin, distal margin, and radial margin), multiple primary cancers, effects of radiation and/or anticancer chemotherapy, lymph node metastasis, distant organ metastasis, and stage.

e.g.: Mt, 5 cm, Type 2, moderately differentiated squamous cell carcinoma, pT3, INFa, ly1, v1, IM0, pPM0, pDM0, pRM0, multiple primary carcinomas (present, two lesions), CRT-grade 2, pN1 (2/30), sM0, fStage III.

**2. Clinical aspects**

2.1. Description of primary tumor

*2.1.1. Number of primary tumors, size and circumferential location*

Maximum length (mm) and orthogonally oriented maximum width (mm), center of circumferential extent, and circumferential ratio of the tumor to the entire esophagus should be described. In addition, the methods used for diagnosis, such as barium X-ray, endoscopy and EUS, should be recorded.

*2.1.2. Tumor location*

2.1.2.1. Anatomical definition of the esophagus

The esophagus is defined anatomically from the esophageal orifice to the esophagogastric junction. The esophageal orifice is at the lower margin of the cricoid cartilage. The identification of the esophagogastric junction (EGJ) will be described later.

2.1.2.2. Anatomical regions (subsites) of the esophagus

The esophagus lies between the hypopharynx and stomach, and can be anatomically divided into the following portions; cervical esophagus (Ce), thoracic esophagus (Te) and abdominal esophagus (Ae). The zone of the esophagogastric junction is divided into the esophageal side (E) and gastric side (G) (Fig. 1-1).^Note^

**Fig. 1-1:**
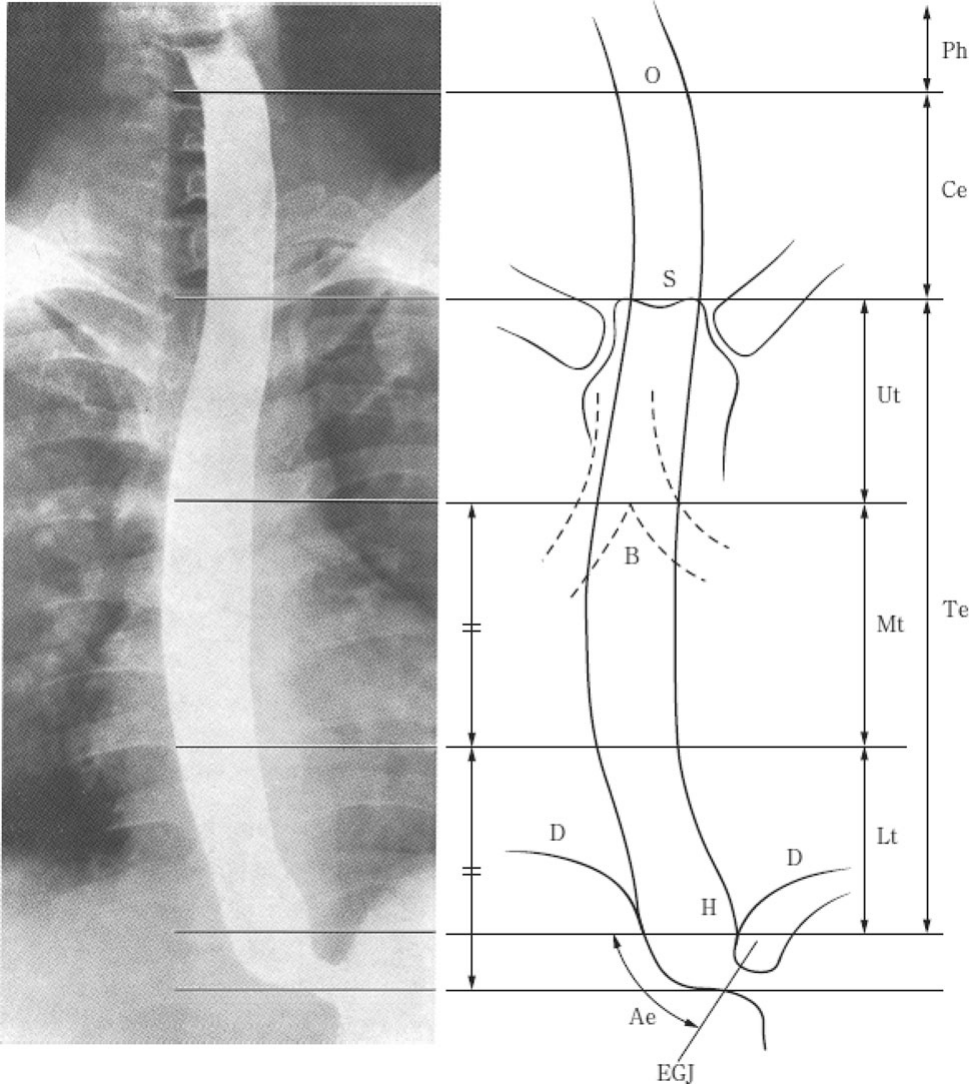
Tumor location. *O* esophageal orifice, *S* superior margin of the sternum, *B* tracheal bifurcation, *D* diaphragm, *EGJ* esophagogastric junction, *H* esophageal hiatus

**Cervical esophagus (Ce)**: This extends from the esophageal orifice to the sternal notch.

**Thoracic esophagus (Te)**: From the sternal notch to the superior margin of the esophageal hiatus.

**Upper thoracic esophagus (Ut)**: From the sternal notch to the tracheal bifurcation.

**Middle thoracic esophagus (Mt)**: The proximal half of the two equal portions between the tracheal bifurcation and the esophagogastric junction.

**Lower thoracic esophagus (Lt)**: The thoracic part of the distal half of the two equal portions between the tracheal bifurcation and the esophagogastric junction.

**Abdominal esophagus (Ae)**: The abdominal part of the distal half of the two equal portions between the tracheal bifurcation and the esophagogastric junction (from the superior margin of the esophageal hiatus to the esophagogastric junction).

- Note: The zone of the esophagogastric junction is defined as the region between 2 cm in esophagus and 2 cm in the stomach from the esophagogastric junction. The abdominal esophagus is included in this zone.

2.1.2.3. Principles of description of tumor location

Describe the tumor location identified by examinations according to the following order of priority: barium X-ray, CT, and endoscopic measurements. Include the distance from the incisor in addition to the tumor location. When the tumor location is uncertain because examinations other than endoscopy have yet to be performed, describe only the distance from the incisor.

When the tumor extends continuously in more than one portion of the esophagus, the main tumor location is that with the deepest tumor invasion. If it is difficult to determine the site of deepest tumor invasion, the portion at the central point of the tumor can be recorded as the main tumor location.

In the case of multiple primary lesions, the locations of the lesions are described in the order of depth of tumor invasion. The deepest lesion is described first. If it is difficult to determine the order of the depth, the description order depends on the size of the area occupied by the lesion. The largest lesion is described first.

e.g.: MtLt, LtAeG.

*2.1.3. Macroscopic tumor type*

2.1.3.1. Principles of tumor type classification

The tumor type classification is based on the macroscopic findings. Radiological and endoscopic classifications are based on the macroscopic classification.

Tumors in which invasion is macroscopically diagnosed to be limited to within the submucosa are classified as superficial type, while tumors in which invasion is diagnosed to extend to the muscularis propria or beyond are classified as advanced type. The superficial type has the prefix ‘0’ and is classified into 0-I, 0-II or 0-III. The advanced type is divided into 4 categories: 1, 2, 3, or 4. When a tumor cannot be classified into any of the 5 (0–4) categories or their combinations, it is classified as 5.

2.1.3.2. Macroscopic classification (Fig. 1-2)^Note 1^

**Fig. 1-2:**
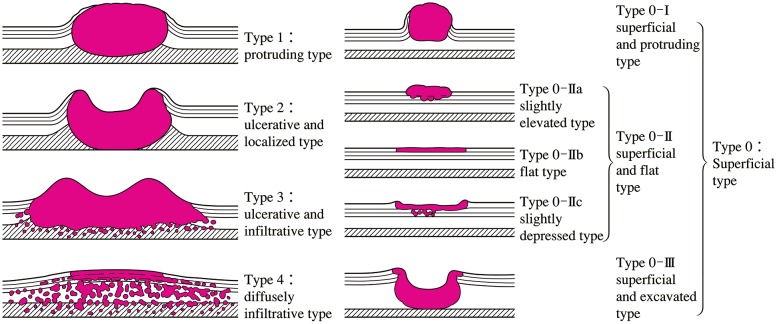
Macroscopic classification (Type 0–4)

Type 0 Superficial type
Type 1 Protruding type
Type 2 Ulcerative and localized type
Type 3 Ulcerative and infiltrative type
Type 4 Diffusely infiltrative type
Type 5 Unclassifiable type
  Type 5a Unclassifiable type without treatment
  Type 5b Unclassifiable type after treatment^Notes 1, 2^

- Note 1: The macroscopic tumor type before chemotherapy and/or radiotherapy is described. Previous treatment is indicated. Cases with minor changes following treatment and which fit the macroscopic tumor type(s) are classified as type 1–4 and cases of major changes are designated as unclassifiable type.
- Note 2: Any former treatment(s) is mentioned before the macroscopic tumor type. e.g.: CT-3, CRT-5b, EMR-0-IIc

2.1.3.3. Subclassification of superficial type (type 0)

Type 0-I Superficial and protruding type
  Type 0-Ip Pedunculated type
  Type 0-Is Sessile (broad based) type
Type 0-II Superficial and flat type
  Type 0-IIa Slightly elevated type
  Type 0-IIb Flat type
  Type 0-IIc Slightly depressed type
Type 0-III Superficial and excavated type

**Other notations**

- Note 1: Combined type: When multiple macroscopic tumor types are mixed in one lesion, it is called a combined type. The wider type is described first and types are connected with +. Double quotation marks (“”) are placed around the macroscopic tumor type that has the deepest tumor invasion. In this case, the main macroscopic tumor type is the deepest one. However, when an advanced type is mixed with a superficial type, the advanced type is described first and double quotation marks are unnecessary.
  e.g.: 0-IIc+“0-Is”, 3+0-IIc.
- Note 2: Superficial spreading type: superficial type 0-II in which the maximal length of the tumor extends 5 cm or more longitudinally. It may be noted additionally in the macroscopic tumor type.

[Reference]

Japanese Society for Esophageal Diseases. Guidelines for the Clinical and Pathologic Studies on Carcinoma of the Esophagus (in Japanese). 8th ed. Kanehara Shuppan, Tokyo, 1992; 34.

*2.1.4. Depth of tumor invasion (T)*

TX Depth of tumor invasion cannot be assessed
T0 No evidence of primary tumor
T1a Tumor invades mucosa^Note 1^(Fig 1-3)
  1. Carcinoma in situ (Tis)
  2. Tumor invades lamina propria mucosae (LPM)
  3. Tumor invades muscularis mucosae (MM)
T1b Tumor invades submucosa (SM)^Notes 2, 3, 4^
  T1b-SM1 Tumor invades the upper third of the submucosal layer
  T1b-SM2 Tumor invades the middle third of the submucosal layer
  T1b-SM3 Tumor invades the lower third of the submucosal layer
T2 Tumor invades muscularis propria (MP)
T3 Tumor invades adventitia (AD)
T4 Tumor invades adjacent structures (AI)^Notes 5, 7^
  T4a Pleura, pericardium, diaphragm, lung, thoracic duct, azygos vein, nerve.
  T4b Aorta (great artery), trachea, bronchus, pulmonary vein, pulmonary artery, vertebral body.

**Fig. 1-3:**
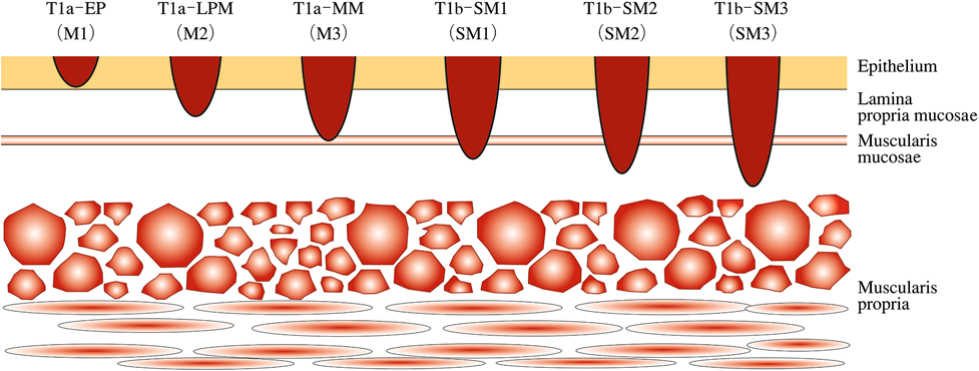
Subclassification for superficial cancer (modified from the guidelines for esophageal cancer treatment)

- Note 1: Early esophageal cancer: T1a can be designated as early cancer of the esophagus regardless of the presence or absence of lymph node or distant organ metastasis. e.g.: early esophageal cancer: T1aNxMx.
- Note 2: Superficial esophageal cancer: T1a and T1b are designated as superficial cancer regardless of lymph node or distant organ metastasis.
  e.g.: superficial esophageal cancer: T1NxMx
- Note 3: Formerly used subclassification of superficial type generally corresponds to the following.
  M1: T1a-EP, M2: T1a-LPM, M3: T1a-MM, SM1: T1b-SM1, SM 2: T1b-SM2, SM 3: T1b-SM3
- Note 4: In endoscopically resected specimens, a tumor invading the submucosa to a depth of 200 μm or less from the lamina muscularis mucosae is classified as T1b-SM1, while a tumor extending more than 200 μm is classified as T1b-SM2, since the distance of the submucosal layer is unknown.
- Note 5: Invaded organs such as the pericardium, aorta, vena cava, trachea, lung, diaphragm, thoracic duct, recurrent laryngeal nerve, azygos vein should be recorded.
  e.g.: T4a (lung).
- Note 6: When a metastatic lymph node additionally invades a surrounding organ other than the esophagus, it should be classified as T4 and recorded as “T4 (metastatic node number-invaded organ)”.
  e.g.: T4b (No.112aoA-Aorta).

2.2. Metastatic lesions from esophageal cancer

*2.2.1. Lymph node metastasis*

2.2.1.1. Naming and numbers of lymph node stations

The names and numbers of lymph nodes are defined as shown in Table 1-5 and Fig. 1-4. The stations of cervical and thoracic lymph nodes are shown in Figs. 1-5, 1-6 and 1-7. The names and numbers of abdominal lymph node stations are defined in the Japanese Classification of Gastric Carcinoma (Table 1-5).

**Fig. 1-4:**
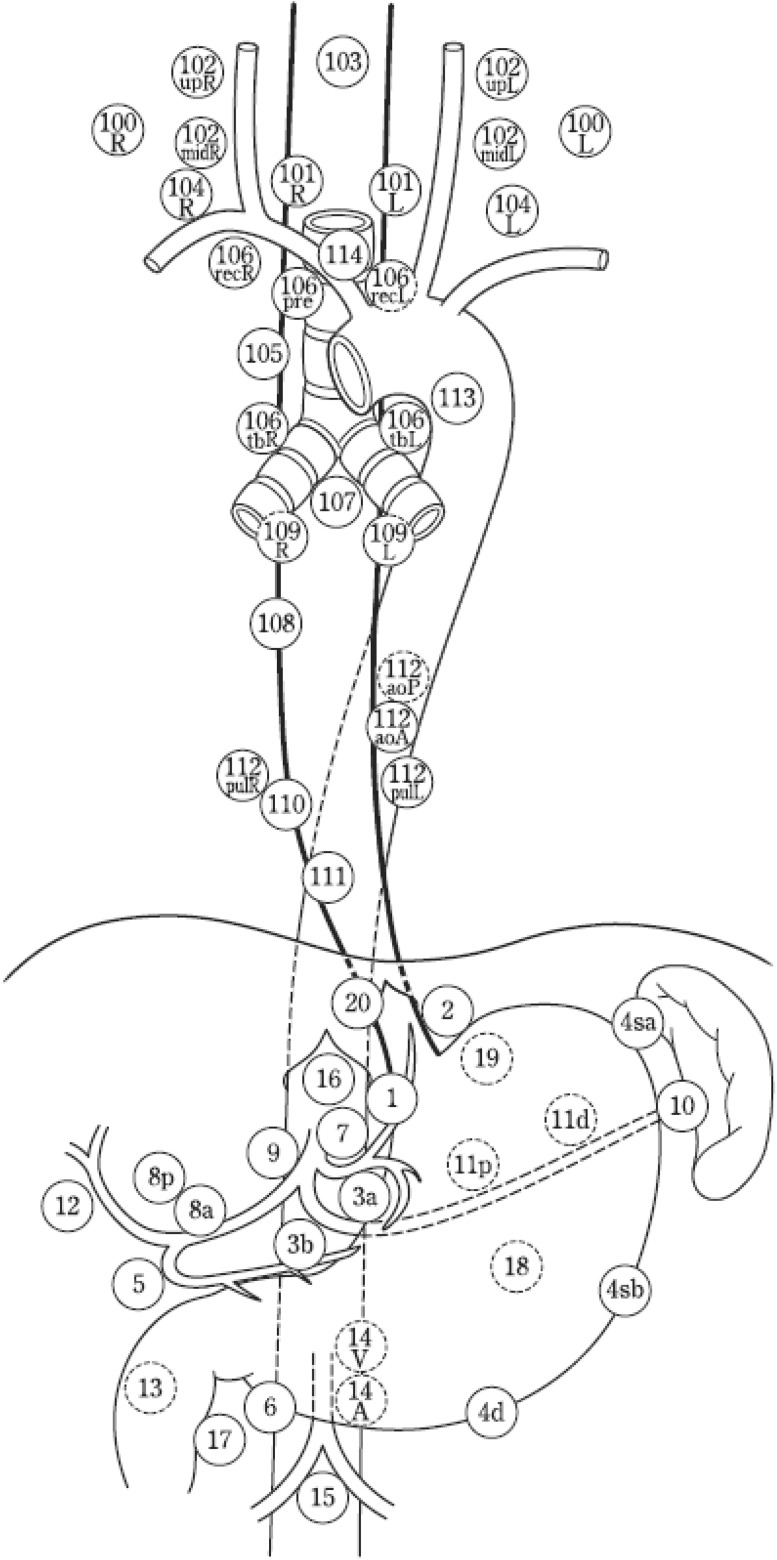
Station numbers of regional lymph nodes

**Fig. 1-5:**
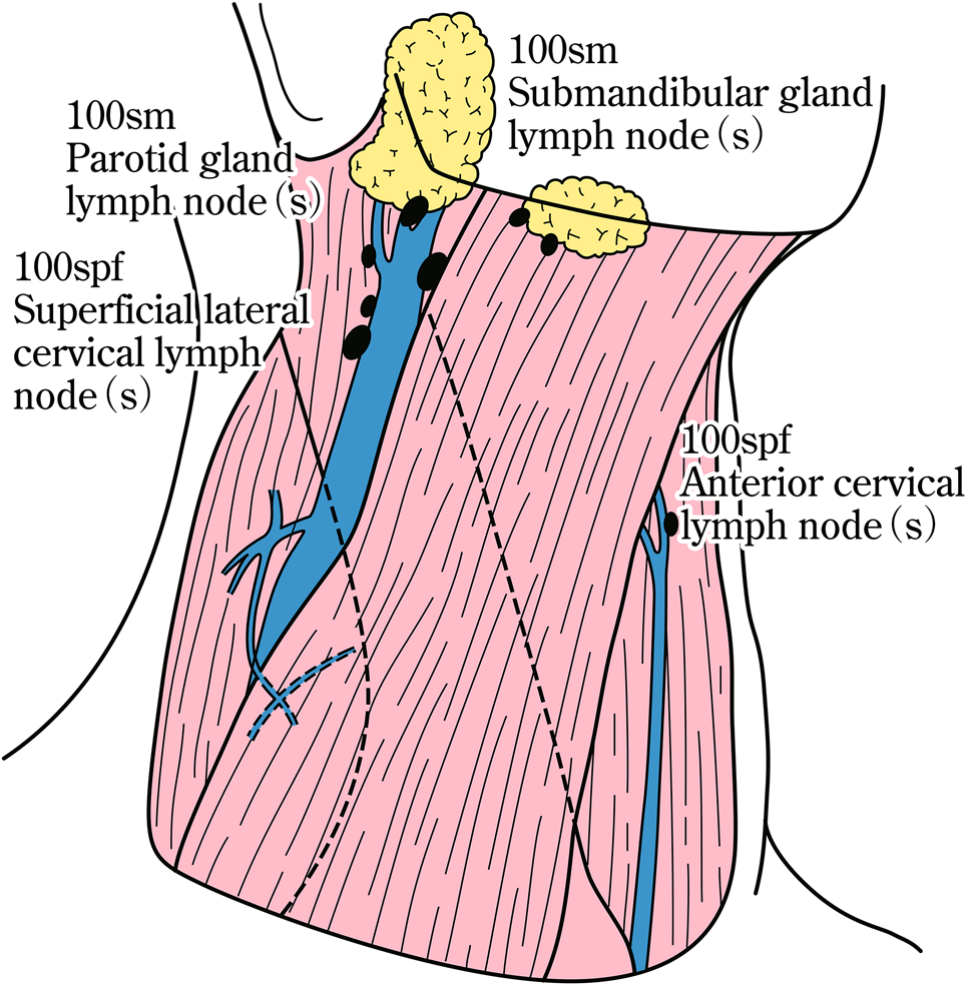
Superficial cervical lymph nodes

**Fig. 1-6:**
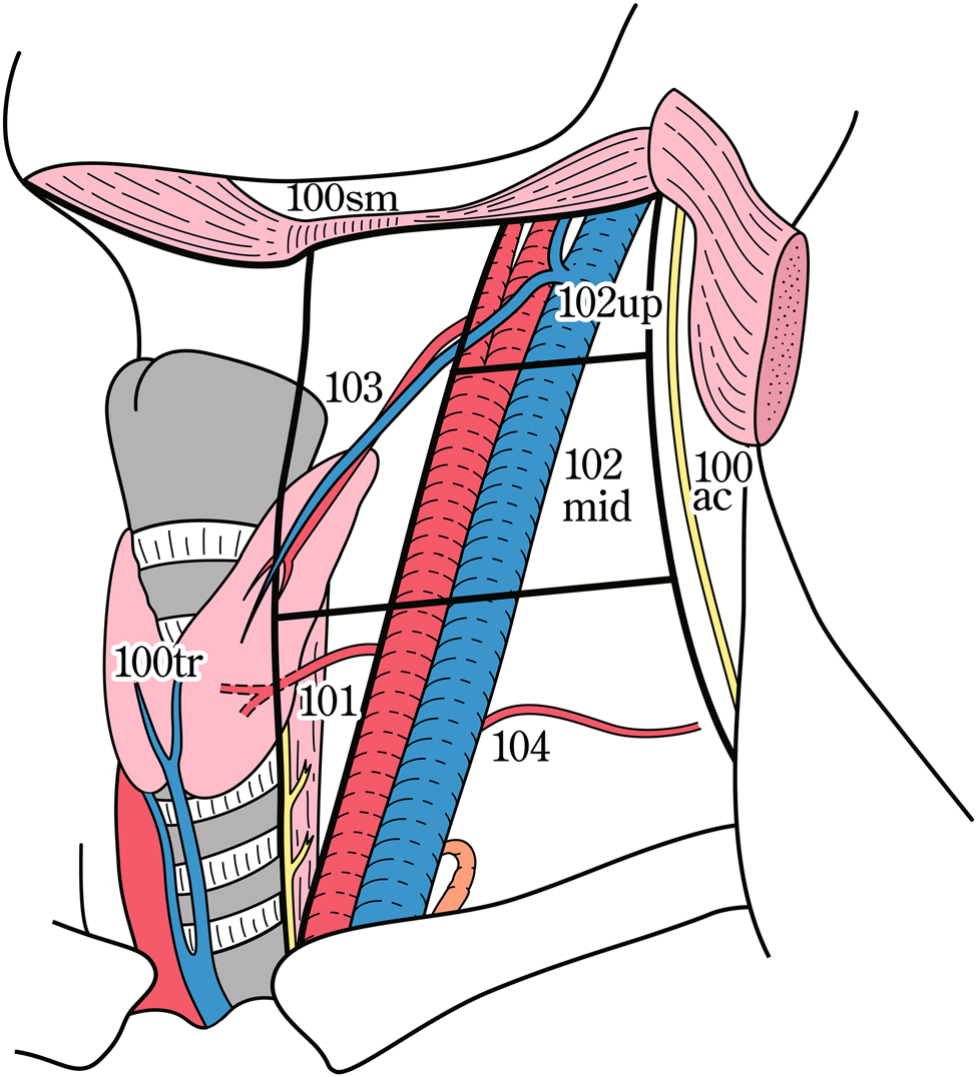
Deep cervical lymph nodes

**Fig. 1-7:**
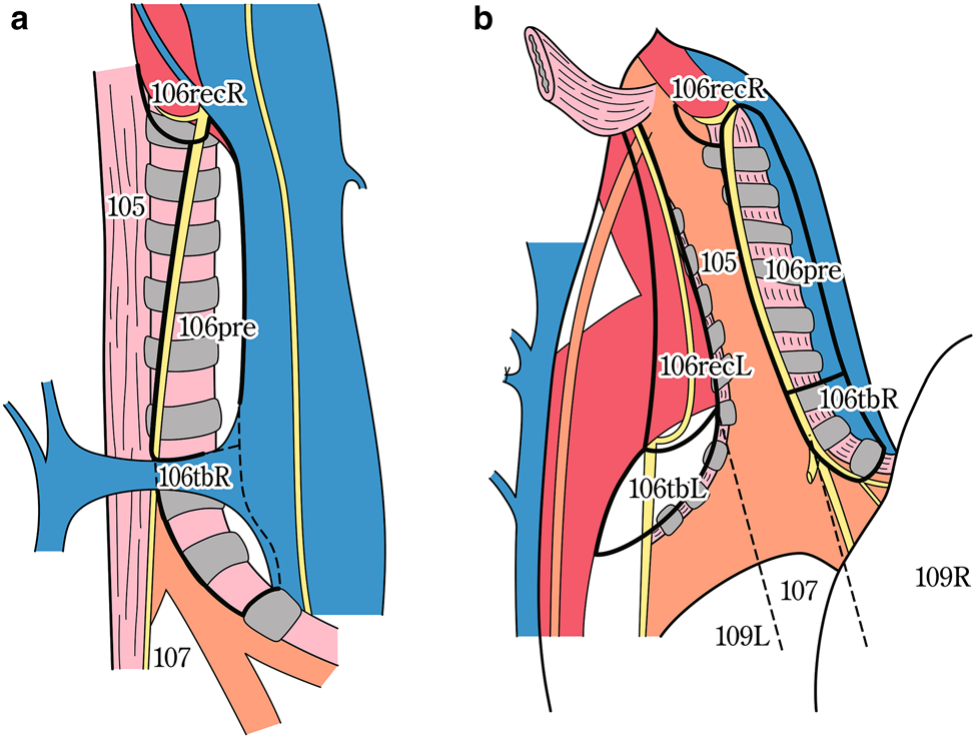
Tracheobronchial lymph nodes (*right view* of the trachea) (posterior view of the trachea)

**Table 1-5:** Numbers and naming of regional lymph nodes

(1) Cervical lymph nodes* (Figs. 1-4, 1-5, 1-6)	
No. 100	Superficial lymph nodes of the neck
No. 100spf	Superficial cervical lymph nodes
No. 100sm	Submandibular lymph nodes
No. 100tr	Cervical pretracheal lymph nodes
No. 100ac	Accessory nerve lymph nodes
No. 101	Cervical paraesophageal lymph nodes
No. 102	Deep cervical lymph nodes
No. 102up	Upper deep cervical lymph nodes
No. 102mid	Middle deep cervical lymph nodes
No. 103	Peripharyngeal lymph nodes
No. 104	Supraclavicular lymph nodes
(2) Thoracic lymph nodes (Figs. 1-4, 1-7)	
No. 105	Upper thoracic paraesophageal lymph nodes
No. 106	Thoracic paratracheal lymph nodes
No. 106rec	Recurrent nerve lymph nodes
No. 106recL	Left recurrent nerve lymph nodes
No. 106recR	Right recurrent nerve lymph nodes
No. 106pre	Pretracheal lymph nodes
No. 106tb	Tracheobronchial lymph nodes
No. 106tbL	Left tracheobronchial lymph nodes
No. 106tbR	Right tracheobronchial lymph nodes
No. 107	Subcarinal lymph nodes
No. 108	Middle thoracic paraesophageal lymph nodes
No. 109	Main bronchus lymph nodes
No. 109L	Left main bronchus lymph nodes
No. 109R	Right main bronchus lymph nodes
No. 110	Lower thoracic paraesophageal lymph nodes
No. 111	Supradiaphragmatic lymph nodes
No. 112	Posterior mediastinal lymph nodes
No. 112aoA	Anterior thoracic paraaortic lymph nodes
No. 112aoP	Posterior thoracic paraaortic lymph nodes
No. 112pul	Pulmonary ligament lymph nodes
No. 113	Ligamentum arteriosum lymph nodes (Botallo lymph nodes)
No. 114	Anterior mediastinal lymph nodes
(3) Abdominal lymph nodes (Fig. 1-4)	
No. 1	Right paracardial lymph nodes
No. 2	Left paracardial lymph nodes
No. 3a	Lesser curvature Lymph nodes along the branches of the left gastric artery
No. 3b	Lesser curvature Lymph nodes along the 2nd branches and distal part of the right gastric artery
No. 4	Lymph nodes along the greater curvature
No. 4sa	Lymph nodes along the short gastric vessels
No. 4sb	Lymph nodes along the left gastroepiploic artery
No. 4d	Lymph nodes along the right gastroepiploic artery
No. 5	Suprapyloric lymph nodes
No. 6	Infrapyloric lymph nodes
No. 7	Lymph nodes along the left gastric artery
No. 8a	Lymph nodes along the common hepatic artery (anterosuperior group)
No. 8p	Lymph nodes along the common hepatic artery (Posterior group)
No. 9	Lymph nodes along the celiac artery
No. 10	Lymph nodes at the splenic hilum
No. 11	Lymph nodes along the splenic artery
No. 11p	Lymph nodes along the proximal splenic artery
No. 11d	Lymph nodes along the distal splenic artery
No. 12	Lymph nodes in the hepatoduodenal ligament
No. 13	Lymph nodes on the posterior surface of the pancreatic head
No. 14	Lymph nodes along the superior mesenteric vessels
No. 14A	Lymph nodes along the superior mesenteric artery
No. 14V	Lymph nodes along the superior mesenteric vein
No. 15	Lymph nodes along the middle colic artery
No. 16	Lymph nodes around the abdominal aorta
No. 16a1	Lymph nodes in the aortic hiatus
No. 16a2	Lymph nodes around the abdominal aorta (from the upper margin of the celiac trunk to the lower margin of the left renal vein)
No. 16b1	Lymph nodes around the abdominal aorta (from the lower margin of the left renal vein to the upper margin of the inferior mesenteric artery)
No. 16b2	Lymph nodes around the abdominal aorta (from the upper margin of the inferior mesenteric artery to the aortic bifurcation)
No. 17	Lymph nodes on the anterior surface of the pancreatic head
No. 18	Lymph nodes along the inferior margin of the pancreas
No. 19	Infradiaphragmatic lymph nodes
No. 20	Lymph nodes in the esophageal hiatus of the diaphragm

The left side (L) and the right side (R) should be distinguished for 101, 102, 104, 106rec, 106tb, 109, and 112pul

- Note: The number of lymph node stations should be recorded using “No.” plus a number.
  e.g.: No.106recR.

[Reference]

Japanese Gastric Cancer Association. Japanese Classification of Gastric Carcinoma. 14th ed. Kanehara Shuppan, Tokyo, 2010.

2.2.1.2. Lymph node groups

Lymph node groups are defined according to the location of the tumor as shown in Table 1-6, Figs. 1-8, 1-9, 1-10, 1-11 and 1-12.

**Fig. 1-8:**
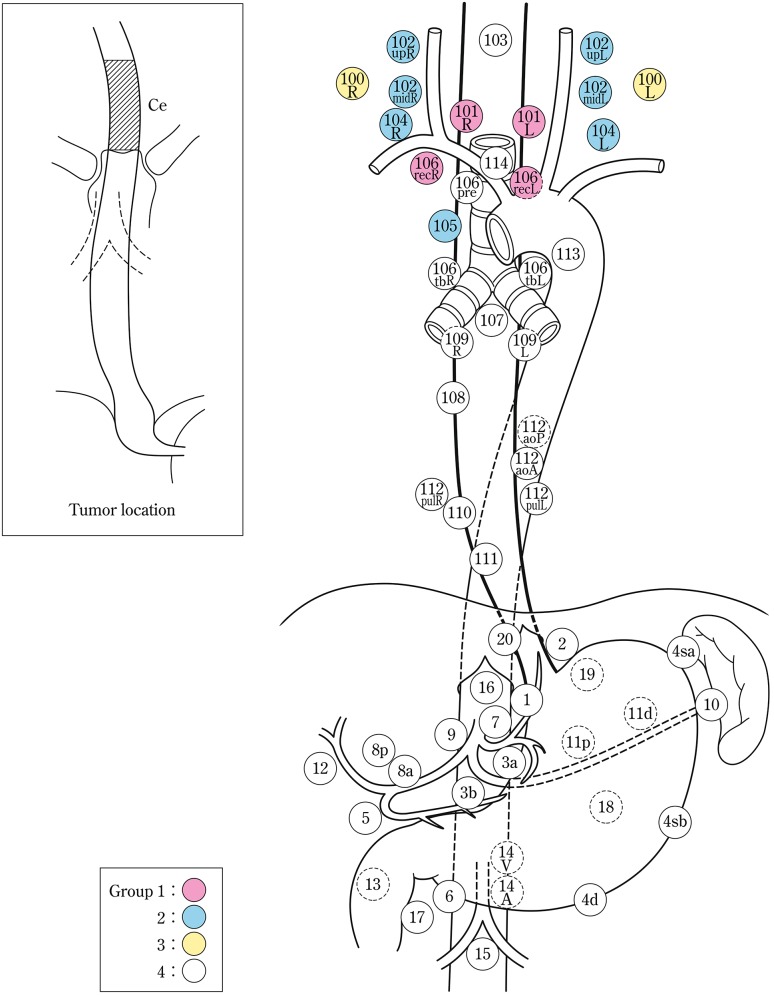
Lymph node groups for tumors located in Ce

**Fig. 1-9:**
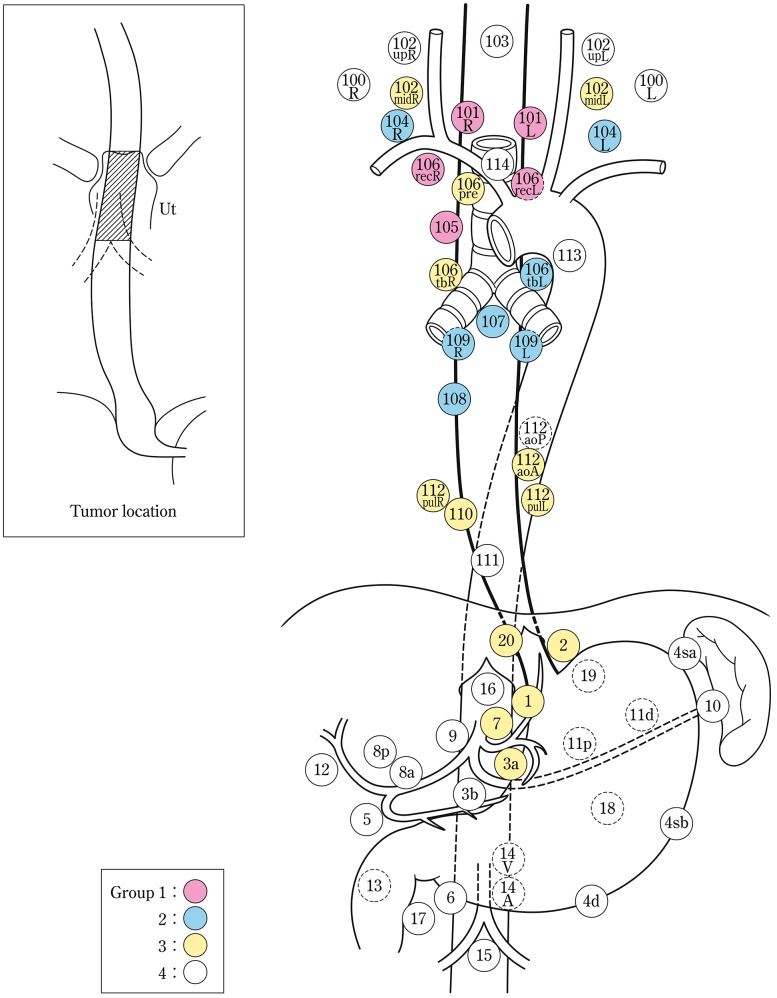
Lymph node groups of tumors located in Ut

**Fig. 1-10:**
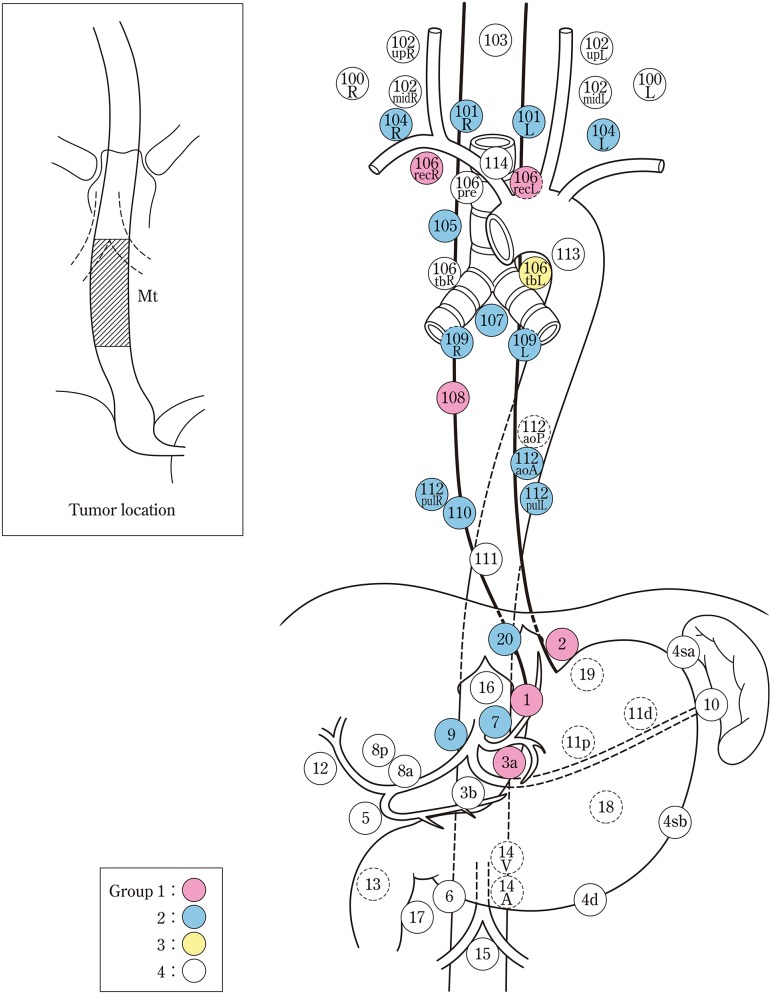
Lymph node groups for tumors located in Mt

**Fig. 1-11:**
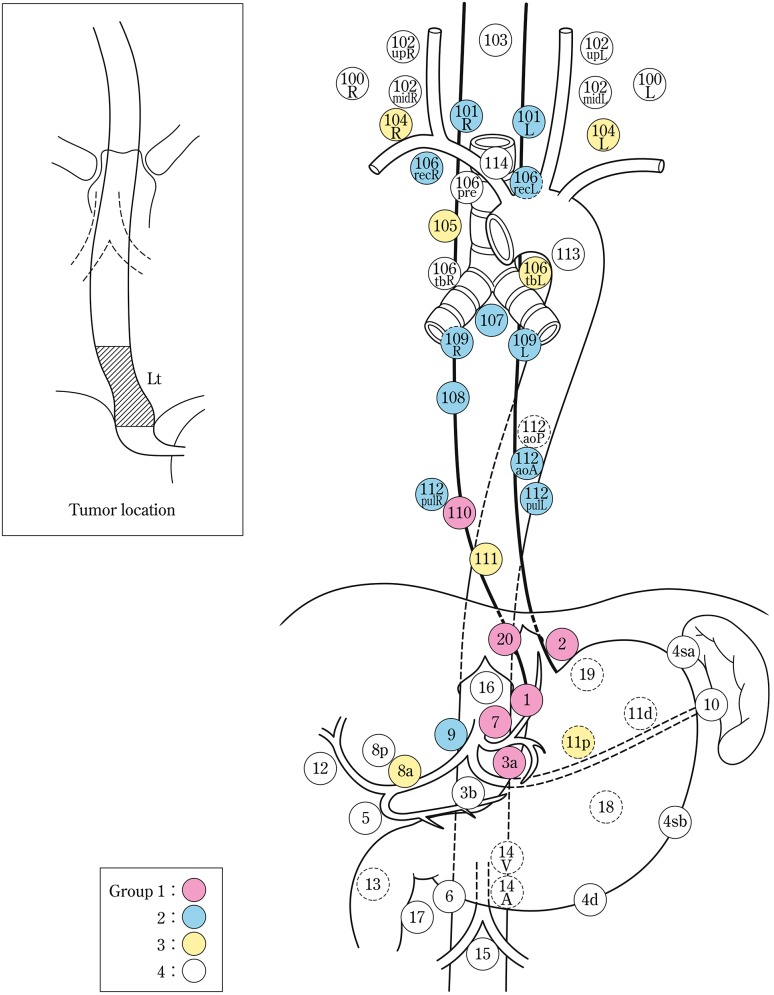
Lymph node groups for tumors located in Lt

**Fig. 1-12:**
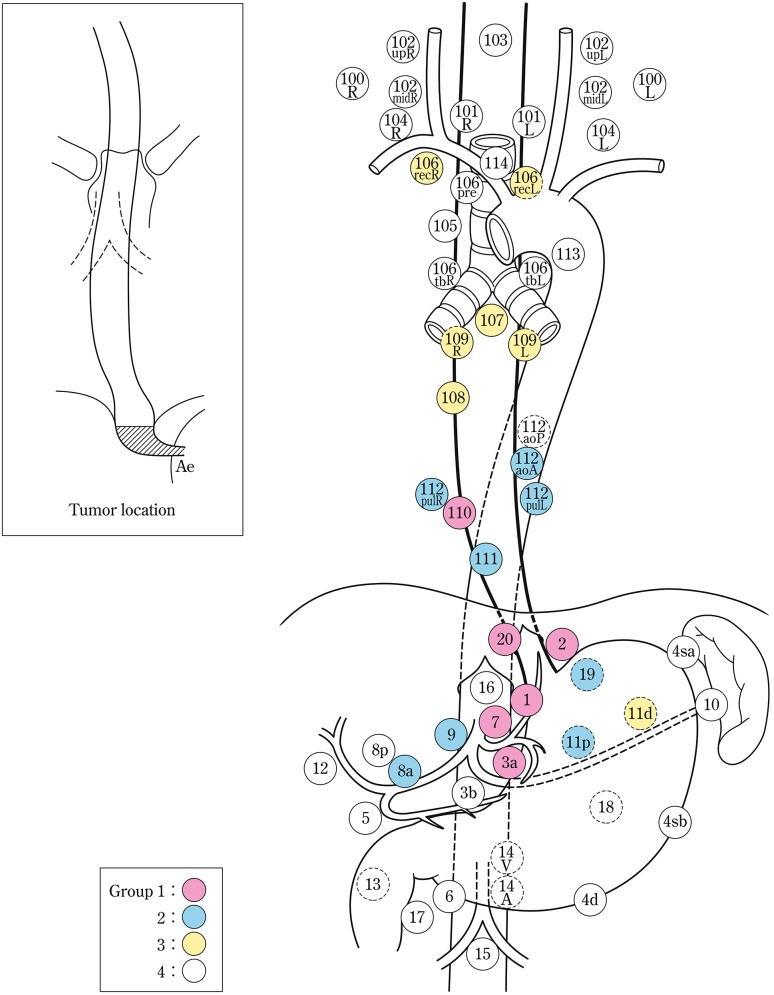
Lymph node groups for tumors located in Ae (EG)

**Table 1-6:** Lymph node groups according to the location of the tumor

Tumor location	Group 1 (N1)	Group 2 (N2)	Group 3 (N3)
Cervical Ce	101, 106rec^a^	102, 104, 105^a^	100
Upper thoracic Ut	101, 105, 106rec	104, 106tbL, 107, 108, 109	102mid, 106pre, 106tbR, 110, 112aoA, 112pul, 1, 2, 3a, 7, 20
Middle thoracic Mt	106rec, 108, 1, 2, 3a	101, 104, 105, 107, 109, 110, 112aoA, 112pul, 7, 9, 20	106tbL
Lower thoracic Lt	110, 1, 2, 3a, 7, 20	101, 106rec, 107, 108, 109, 112aoA, 112pul, 9	104, 105, 106tbL, 111, 8a, 11p
Abdominal Ae	110, 1, 2, 3a, 7, 20	111, 112aoA, 112pul, 8a, 9, 11p, 19	106rec, 107, 108, 109, 11d

Nodes other than N1 through N3 are expressed as N4

^a^Limited to the area which can be dissected from the cervical incision

- Note: In deciding the lymph node group of multiple esophageal cancers and widely extending esophageal cancer, the location of the deepest tumor invasion takes precedence in documentation.

2.2.1.3. Grading of lymph node metastasis (N)

NX: Lymph node metastasis cannot be assessed
N0: No lymph node metastasis
N1: Metastasis involving only Group 1 lymph nodes
N2: Metastasis to Group 2 lymph nodes, regardless of involvement of Group 1 lymph nodes
N3: Metastasis to Group 3 lymph nodes, regardless of involvement of Group 1 or 2 lymph nodes
N4: Metastasis to distant (Group 4) lymph nodes, regardless of whether any other group(s) of regional lymph nodes are involved or not

- Note: Extralymph node metastasis (tumor nodule) is included within N.

*2.2.2. Distant organ metastasis (M)*

MX: Distant organ metastasis cannot be assessed
M0: No distant organ metastasis
M1: Distant organ metastasis

- Note 1: Organs with metastasis should be recorded in parentheses.
  e.g.: M1 (lung), M1 (liver, stomach).
- Note 2: Pleural, peritoneal, and pericardial dissemination should be recorded as M1.

2.3. Stage (Table 1-7)

**Table 1-7:**
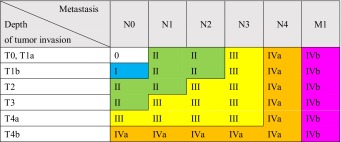
Stage

*T4a* pleura, pericardium, diaphragm, lung, thoracic duct, azygos vein, nerve

*T4b* aorta (large vessel), trachea, bronchus, pulmonary vein, pulmonary artery, vertebra

The stage should be recorded based on the following TNM stage classification.

e.g.: T2N2M0, Stage III.

2.4. Multiple primary cancers

Multiple primary cancers of the esophagus:

The term “multiple primary cancers of the esophagus” is used to refer to the presence of two or more primary esophageal cancers.

- Note: Descriptions of the locations of multiple primary cancers of the esophagus should be made according to the order of the depth of tumor invasion (deeper to shallower), inserting “/” between the abbreviations for the location of each lesion; the total number of lesions should also be recorded in parentheses.
  e.g.: MtUt/Lt/Lt (3 lesions).

Multi-organ primary cancers including the esophagus:

The term “multi-organ primary cancers including the esophagus” is used to refer to the presence of one or more primary malignant diseases other than esophageal cancer in a patient with primary esophageal cancer.

Multiple primary cancers including the esophagus:

The term “multiple primary cancers including the esophagus” indicates the concept combining both “multiple primary cancers of the esophagus” and “multi-organ primary cancers including the esophagus”.

- Note 1: In cases with multi-organ primary cancers including the esophagus, organs other than the esophagus should be specified in parentheses.
- Note 2: Whether the multiplicity is synchronous or metachronous should be recorded.
  e.g.: Multi-organ primary cancers: stomach (synchronous).

**3. Surgical aspects**

3.1. Handling of the resected specimen

The resected esophagus should be cut and opened along the longitudinal line on the side opposite to the lesion. The opened esophagus should be gently stretched longitudinally and fixed so that the length of the specimen becomes similar to its size in vivo. The specimen should be treated with iodine solution after fixation in order to accurately describe the macroscopic findings. This is particularly important in superficial carcinoma. Photographic recording is recommended for both fresh and fixed specimens.

3.2. Description of surgical findings and macroscopic findings of primary tumor

Operative findings should be identified in the record putting “s” in front of each factor.

e.g.: sT2, sStageII.

3.2.1. Tumor size (Fig. 1-13)

**Fig. 13:**
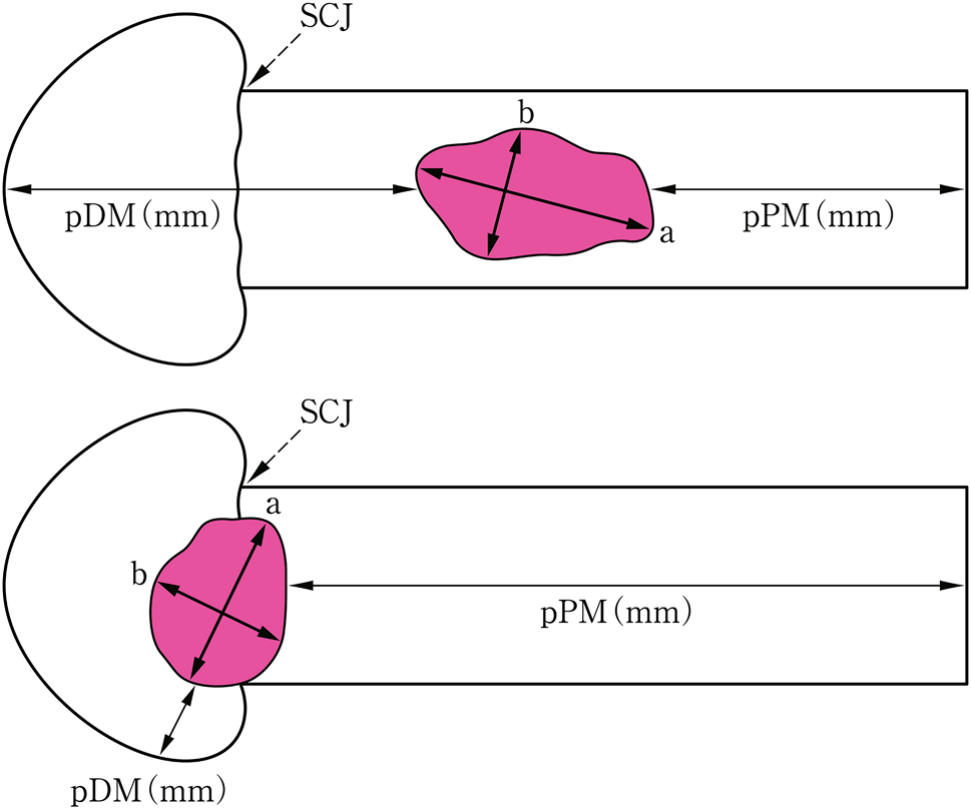
Tumor size and the distance from resection margin to tumor. *a* Greatest longitudinal dimension (mm). *b* Greatest transverse dimension (mm)

The greatest longitudinal dimension in millimeters and the greatest transverse (at 90° to the longitudinal tumor axis) dimension in millimeters: a × b (mm)

*3.2.2. Distance from surgical margin to the tumor (Fig.* 1-13 *)*

Proximal (oral) margin (PM) (mm)

Distal (anal) margin (DM) (mm)

*3.2.3. Macroscopic tumor type*

The macroscopic appearance of tumors before and after fixation can be different. Under such circumstances, the macroscopic tumor type should be described according to pre-fixation observations, and the pathological tumor type should be described based on the post-fixation findings. Pathological tumor types can be classified referring to the cross-sectional observation. Macroscopic tumor types should be determined regardless of microscopic depth of tumor invasion.

- Note: The presence of preoperative chemotherapy and radiotherapy should be recorded with the macroscopic tumor type.

*3.2.4. Surgical margin*

3.2.4.1. PM: Proximal (oral) margin

PMX: Proximal margin cannot be assessed
PM0: No evidence of tumor invasion
PM1: Tumor invasion

3.2.4.2. DM: Distal (anal) margin

DMX: Distal margin cannot be assessed
DM0: No evidence of tumor invasion
DM1: Tumor invasion

- Note: The distance from the resection margin to tumor should be recorded in millimeters for PM0 and DM0 specimens.

*3.2.5. RM: Radial margin*

RMX: Radial margin cannot be assessed
RM0: No evidence of tumor invasion
RM1: Tumor invasion

- Note: The radial margin is the surgical margin in the radial direction, i.e., the outer surface of the surgical dissection plane.

3.3. Intramural metastasis and multiple cancers in the esophagus

*3.3.1. IM: Intramural metastasis*

Metastatic lesions in the esophageal, pharyngeal, or gastric wall macroscopically (clearly) separate from the primary tumor should be recorded as IM, and the number of such lesions should be described.

IMX: Intramural metastasis cannot be assessed
IM0: No intramural metastasis
IM1: Intramural metastasis

- Note: IM in the gastric wall should be recorded as “IM1-St”. It is classified as organ metastasis (M1).

*3.3.2. Multiple cancers of the esophagus*

Multiple cancers are two or more primary cancer lesions separate from each other. Multiple cancers and IM should be clearly differentiated in the description.

3.4. Lymph nodes

*3.4.1. Preparation of resected lymph nodes for pathological examination*

Surgically dissected lymph nodes are classified according to the definition of regional lymph nodes, given individual names or numbers and sent to pathologists. The lymph nodes dissected en bloc with the esophagus should be isolated from the specimen before fixation.

*3.4.2. Grading of lymph node metastasis (N)*

The surgical diagnosis of the grading of lymph node metastasis (sN) should be made comprehensively with intraoperative findings of macroscopic observation, imaging examinations, immediate pathological diagnosis with frozen section, and macroscopic findings obtained during postoperative preparation.

*3.4.3. Lymph node dissection (D)*

3.4.3.1. Field of lymph node dissection

Three-field dissection: Dissection of cervical, thoracic and abdominal lymph nodes through cervical, thoracic and abdominal approaches, respectively^Note^
Two-field dissection: Dissection of thoracic and abdominal lymph nodes through thoracic and abdominal approaches, respectively.
  Dissection of cervical and abdominal lymph nodes through cervical and abdominal approaches, respectively.
  Dissection of cervical and thoracic lymph nodes through cervical and thoracic approaches, respectively.

One-field dissection: Dissection of a single field of cervical, thoracic and abdominal lymph nodes through cervical, thoracic or abdominal approaches, respectively.

- Note: The term “three-field dissection” should not be applied when only the cervical paraesophageal nodes (101R, 101 L) are dissected in the neck.

3.4.3.2. Extent of lymph node dissection (D)

DX: Extent of lymph node dissection cannot be assessed.
D0: No or incomplete dissection of Group-1 lymph nodes.
D1: Complete dissection of Group-1 lymph nodes, but no or incomplete dissection of Group-2 lymph nodes.
D2: Complete dissection of Group-1 and Group-2 lymph nodes, but no or incomplete dissection of Group-3 lymph nodes.
D3: Complete dissection of Group-1, Group-2 and Group-3 lymph nodes

3.5. Distant organ metastasis (M)

Surgical findings of distant organ metastasis (sM) should be determined through comprehensive consideration of operative macroscopic findings, intraoperative imaging examinations such as intraoperative ultrasound examination, macroscopic observation of resected specimen, and intraoperative immediate pathological diagnosis with frozen section. Whether the distant organ metastasis was resected or not should be recorded.

*3.6. Residual tumor (R)* ^*Note 1*^ (Fig. 1-14)

RX: Presence of residual tumor cannot be assessed.
R0: No residual tumor.
R1: Microscopic residual tumor^Note 2^
R2: Macroscopic residual tumor^Note 3^

- Note 1: The postoperative state of both primary tumor and metastatic lesions should be evaluated.
- Note 2: This refers to the presence of a tumor on the surgical margin of the resected specimen that was identified upon microscopic examination.
- Note 3: This refers to a macroscopically obvious residual tumor.

3.7. Curativity (Cur) (Table 1-8)

**Table 1-8:** Surgical curativity

	Stage	N and D	PM, DM, RM	R
Cur A	Stage 0, I, II, III	D > N	PM0, DM0, RM0	R0
Cur B	Neither Cur A nor Cur C			
Cur C	Residual tumor assessed by surgical (macroscopic) findings, R2

Cur A: Complete removal of the tumor is strongly believed.
  sStage 0–III, and sR0, and sD > sN

Cur B: Neither Cur A nor Cur C R1.
  sStage IVa, sStage IVb or sD≦sN, but R0 was achieved with resection of a T4b tumor or complete removal of metastatic tumor (M1) or lymph nodes.

Cur C: Residual tumor.
  R2, i.e., M1 evident residual tumor in distant organ(s) (M1), lymph nodes, or surgical margin(s) (PM1, DM1, RM1).

**Fig. 1-14:**
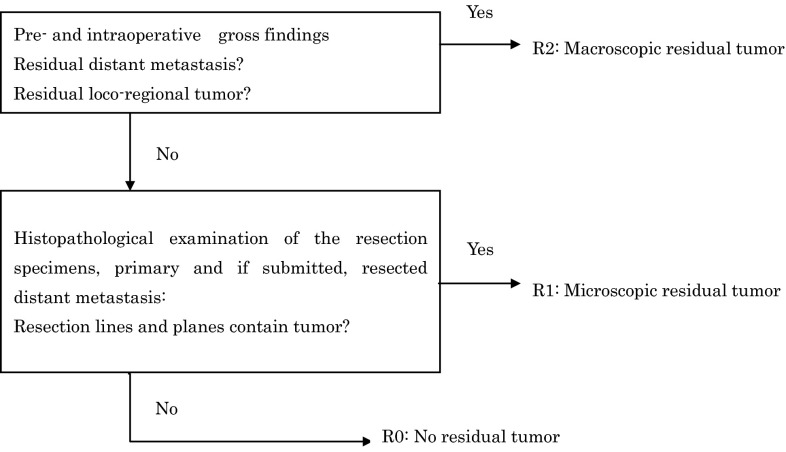
Curativity

**4. Pathological findings**

4.1. Handling of the surgically resected specimens (Fig. 1-15)

Before cutting the resected esophagus, the formalin-fixed specimen should be treated with iodine solution to confirm the unstained area. Rinsing the sample with tap water for at least 1 h can result in a good staining condition. To increase the contrast between stained and unstained areas, the sample should be treated with a relatively low concentration (0.1–0.5%) of iodine solution for a long time.

The resected specimen should be cut parallel along the long axis of the esophagus. Whole step sections are made in superficial type cancer. One representative section of an advanced tumor at the site of deepest invasion, parallel or perpendicular to the esophagus should be blocked and used for microscopic examination. Schemas or photographs of the sites of cut sections should be preserved.

4.2. Description of pathological findings

The p (pathology) mark is prefixed to the pathological findings except for vascular invasion as follows.

e.g.: p0-Is, pType 2, pT2, pStagedII.

*4.2.1. Histological classification *

**Fig. 1-15:**
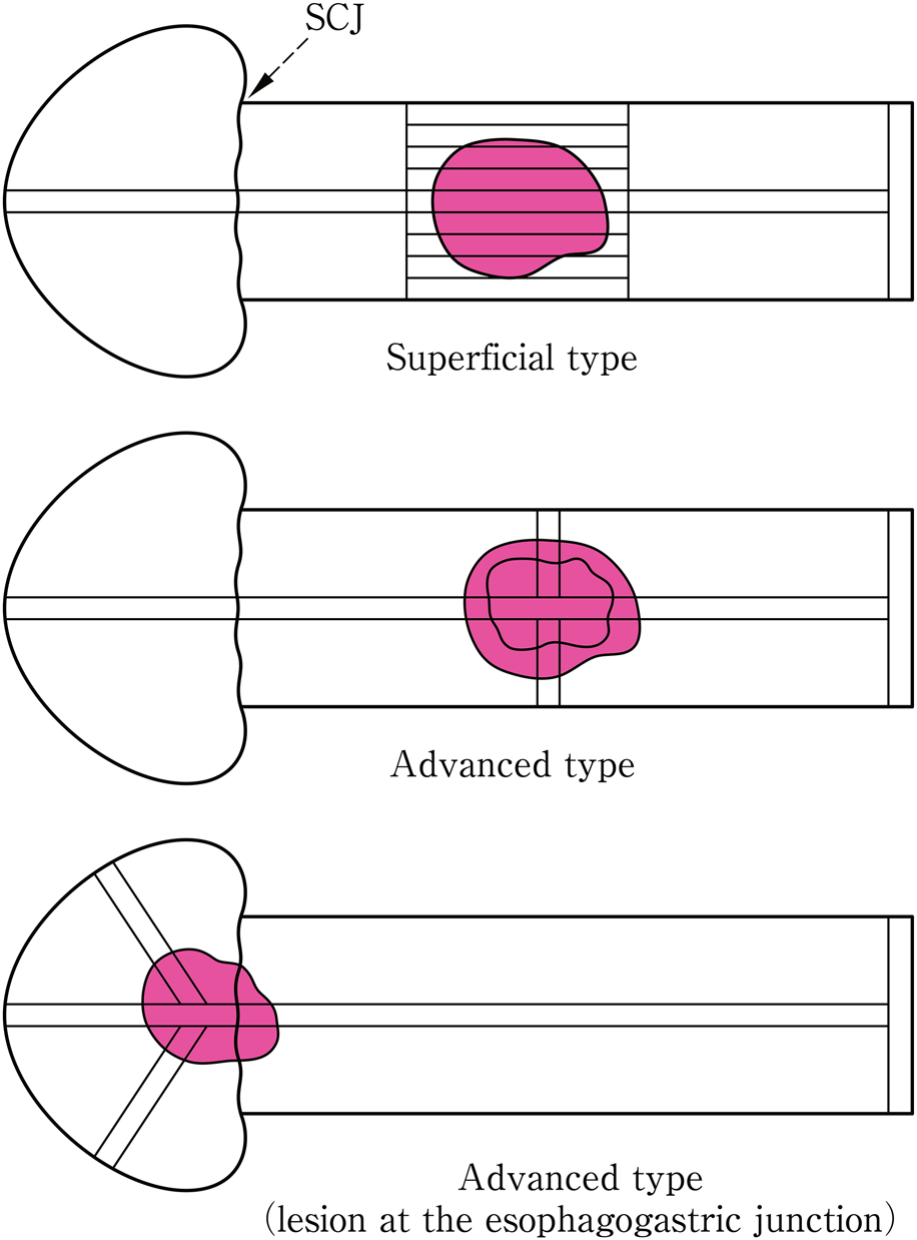
How to cut surgically resected specimens

4.2.1.1. Benign epithelial neoplasms^Note 1^

1. Squamous cell papilloma
2. Adenoma
3. Others

4.2.1.2. Intraepithelial neoplasias^Note 2–7^

1. Squamous intraepithelial neoplasia

4.2.1.3. Malignant epithelial neoplasms

1. Squamous cell carcinoma
  - Well differentiated
  - Moderately differentiated
  - Poorly differentiated
2. Basaloid (-squamous) carcinoma
3. Carcinosarcoma
4. Adenocarcinoma
  - Well differentiated
  - Moderately differentiated
  - Poorly differentiated
5. Adenosquamous carcinoma
6. Mucoepidermoid carcinoma
7. Adenoid cystic carcinoma
8. Neuroendocrine cell tumor^Note 8^
  - Neuroendocrine tumor (NET) G1 or G2
  - Neuroendocrine carcinoma
9. Undifferentiated carcinoma
10. Others

4.2.1.4. Non-epithelial tumors

1. Smooth muscle tumor
2. Gastrointestinal stromal tumor (GIST)
3. Neurogenic tumor
  Schwannoma, neurofibroma, granular cell tumor.
4. Others
  Hemangioma, lymphangioma, lipoma, etc.

4.2.1.5. Lymphoid tumors

The definition is according to the WHO classification.

[Reference]

Swerdlow SH, Campo E, Harris NL, et al. WHO Classification of Tumours of Haematopoietic and Lymphoid Tissues, fourth edition. IARC, Lyon, 2008.

4.2.1.6. Other malignant tumors

1. Malignant melanoma
2. Others

4.2.1.7. Tumor-like lesions

Ectopic gastric mucosa

Heterotopic sebaceous gland

Cowden disease

Glycogenic acanthosis

Fibrovascular polyp

- Note 1: Squamous papilloma is not a true neoplasia, but reactive hyperplasia.
- Note 2: Adenocarcinoma and a tumor-like lesion arising from Barrett mucosa are excluded. The classification of adenocarcinoma in Barrett esophagus is the same as that in the Japanese Classification of Gastric Carcinoma.
- Note 3: According to the WHO classification, high-grade intraepithelial neoplasia cannot be diagnosed as carcinoma because of the absence of invasion. In the 11th edition, however, intraepithelial squamous cell carcinoma (pT1a-EP carcinoma) or squamous cell carcinoma in situ can be diagnosed when cellular and structural atypia are sufficient to suggest malignancy. The 10th edition mentioned that low-grade intraepithelial neoplasia might contain basal-type squamous cell carcinoma. When such lesions are distributed in the lower half of the epithelium and are sufficiently atypical to suggest malignancy, the lesion can be diagnosed as squamous cell carcinoma according to the classification of the 11th edition.
- Note 4: Most “squamous intraepithelial neoplasias” according to the definition of the 11th edition are endoscopically or macroscopically recognized as a “small unstained or tan-stained area”. The lesion may be solitary or multiple.
- Note 5: According to the definition of the 11th edition, intraepithelial neoplastic lesion without atypia sufficient to suggest malignancy is termed as squamous intraepithelial neoplasia. Thus, intraepithelial neoplasia does not include squamous cell carcinoma in situ. Please be careful to note the differences in the definitions of intraepithelial neoplasia between the 10th and 11th editions. A two-tier subclassification of intraepithelial neoplasia (low grade and high grade) is not used in the 11th edition. In making a diagnosis of intraepithelial neoplasia based on a biopsy specimen, the inclusion of one of the following comments is recommended: “follow-up is needed”, “re-biopsy after a short time period should be recommended”, or “immediate re-biopsy is strongly recommended because of suspicious carcinoma”.
- Note 6: When the determination of a biopsy specimen as “neoplastic” or “reactive” is difficult, the specimen should not be diagnosed as “intraepithelial neoplasia”, but rather as “atypical epithelium” or “atypical epithelium, indefinite for neoplasia”. For clinicians, the inclusion of instructions such as the need for a re-biopsy is recommended.
- Note 7: Squamous cell carcinoma, which is limited to within the epithelial layer without invasion, is different from squamous intraepithelial neoplasia. Squamous cell carcinoma in situ is equal to squamous cell carcinoma with a depth of pT1a-EP.
- Note 8: Neuroendocrine tumor and neuroendocrine carcinoma are formally classified as carcinoid tumor and endocrine cell carcinoma, respectively. These terms have been adopted according to the WHO classification. In Japan, however, “endocrine cell carcinoma” is considered to be the correct term, since endocrine cells in the gastrointestinal tract originate from gastrointestinal stem cells.

*4.2.2. Depth of tumor invasion (pT)* ^*Note 1–7*^

- Note 1: Intraductal spreading of cancer is categorized as pT1a-EP, and if the tumor invades beyond the duct of the esophageal gland, the depth of tumor invasion is defined as the layer presenting extraductal invasion of cancer.
- Note 2: The vertical depth of submucosal invasion is measured from the muscularis mucosae, and the depth is recorded in parentheses.
  e.g.: pT1b-SM2 (400 μm).
- Note 3: The depth of tumor invasion is defined histologically as the point of deepest direct invasion by the primary tumor. Vascular invasion within the confines of the primary tumor should be regarded as the depth of direct tumor invasion. However, when vascular invasion is found outside the confines of the primary tumor, the depth of such invasion should be specified in parentheses after the depth of direct invasion. For example, when a primary tumor has invaded into the submucosa (pT1b) but lymphatic invasion is found in the muscularis propria outside the main tumor, this is designated as pT1b (ly-T2).
- Note 4: Cancer that has macroscopically invaded adjacent organ(s) (sT4) and histologically diagnosed malignant tissue recognized on the surgical radial margin (pRM1) are categorized as pT4.
- Note 5: Direct invasion of tumor from lymph node metastasis to the adjacent organ(s) is categorized as pT4.
  e.g.: Direct invasion from No.108 lymph node metastasis to the lung: pN1 (108-lung) T4a
- Note 6: In determining the depth of invasion of an advanced cancer after preoperative treatment, both the depth of invasion by residual tumor and the estimated depth of tumor invasion prior to treatment should be considered. The type of adjuvant therapy (RT-, CT-, CRT-, EMR-), depth of invasion by the residual tumor, and estimated depth of tumor invasion prior to treatment should be specified in the given order, with the last item in parentheses. e.g.: RT-pT1b (T4).
- Note 7: If no residual tumor is found in an entire specimen after preoperative treatment, the designation should be pT0, and its stage is recognized as the same as T1a.
  e.g.: CRT-pT0 (T3), N0, M0, CRT-pStage 0.

*4.2.3. Infiltrative growth pattern (INF)*

The growth and infiltrative pattern of tumor can be classified into one of the following three types, with regard to the predominant pattern observed at tumor margins.

INFa (expansive type): Expansive growth of tumor nests with a well-demarcated border from surrounding tissue.
INFb (intermediate type): Intermediate growth pattern, between INFa and INFc.
INFc (infiltrative type): Infiltrative growth of tumor nests with an ill-defined border from surrounding tissue.

*4.2.4. Vascular invasion (ly/v)* ^*Note 1*^

- Note 1: Indefinite for determination of lymphatic or venous invasion is described as ly/v.

4.2.4.1. Lymphatic invasion (ly)^Note 1^

ly0: None
ly1: Slight
ly2: Moderate
ly3: Severe^Notes 2–3^

- Note 1: Examination using immunohistochemical staining with an anti-D2-40 antibody should be described. e.g.: ly1 (D2-40).
- Note 2: Carcinomatous lymphangiosis in distant organ(s) is categorized as M1.
- Note 3: A tumor mass found in the thoracic duct is described as positive lymphatic invasion.

4.2.4.2. Venous invasion (v)^Note 1^

v0: None
v1: Slight
v2: Moderate
v3: Severe

- Note 1: Examination by elastic fiber staining methods should be described.
  e.g.: v1 (Elastica van Gieson), v2 (Victoria blue).

*4.2.5. Intramural metastasis (pIM)*

*4.2.6. Distance from Surgical margin*

4.2.6.1. Proximal and distal margin (pPM, pDM)^Note^

- Note: The distance from surgical margin to tumor edge in pPM0 or pDM0 is measured in histological specimens (mm).

4.2.6.2. Radial margin (pRM)

*4.2.7. Multiple primary cancers*

Present (number of lesions).

Absent.

- Note: A lesion with a histological type different from that of the main tumor or an isolated lesion with an intraepithelial component is recognized as another primary cancer, and the patient is classified as having multiple primary cancers.

*4.2.8. Others*

1. Metastatic or invasive cancer from other organs.
2. Co-existing tumor.
  Leiomyoma, etc.
3. Other non-neoplastic lesions.

Barrett esophagus, Achalasia, etc.

*4.2.9. Pathological criteria for the effects of radiation and/or chemotherapy (Fig.* 1-16 *)*

**Fig. 1-16:**
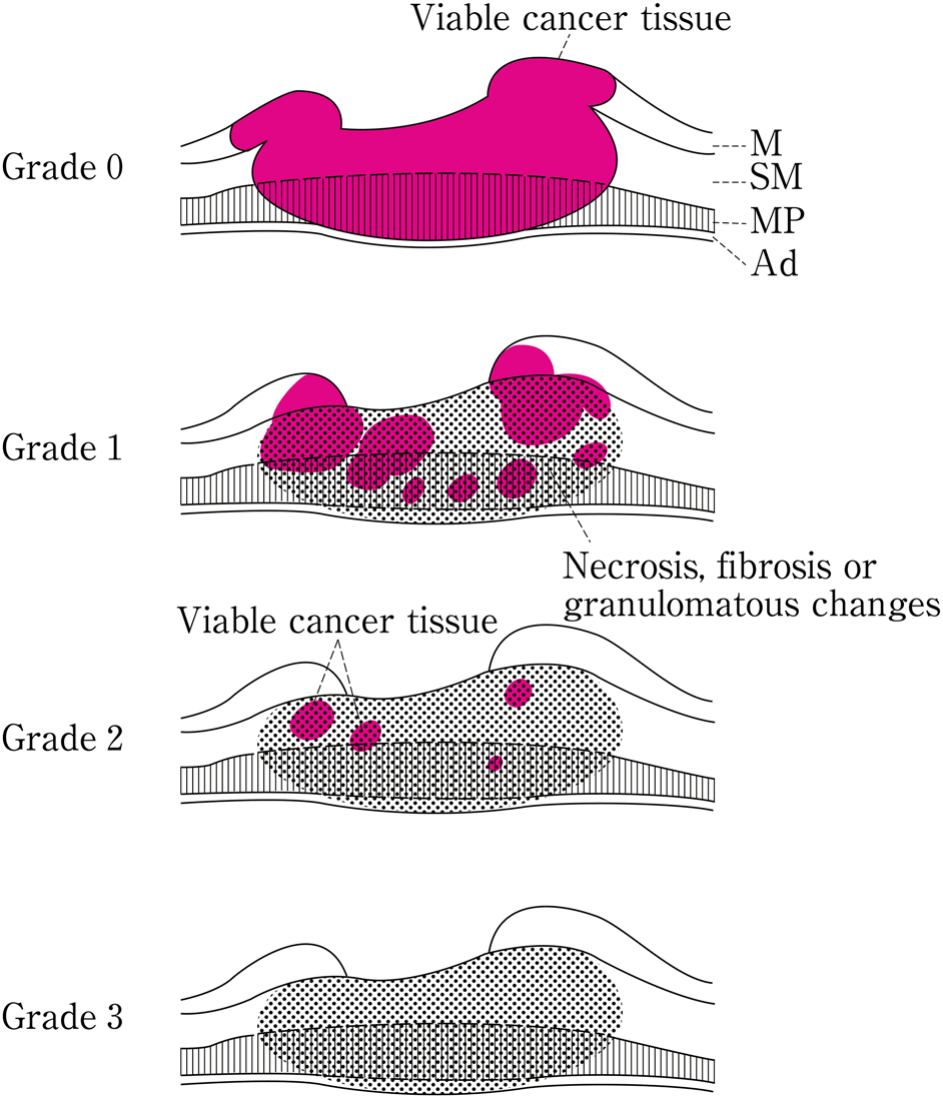
Histological efficacy of chemotherapy and/or radiotherapy

In cases of preoperative radiation and/or chemotherapy, the radiation dose and method of administration, type and dose of chemotherapy, and time interval between preoperative therapy and surgical resection of the tumor are described. In cases of preoperative treatment, all the specimens in which the primary tumor is macroscopically possibly present should be examined histologically.

- Grade 0: ineffective
  - No recognizable cytological or histological therapeutic effect.
- Grade 1: slightly effective
  - Apparently viable cancer cells (including cells having eosinophilic cytoplasm with vacuolation and swollen nuclei) account for 1/3 or more of tumor tissue, but there is some evidence of degeneration of cancer tissue or cells.
    -
      - Grade 1a: Viable cancer cells accounting for 2/3 or more tumor tissue.
      - Grade 1b: Viable cancer cells accounting for 1/3 or more, but less than 2/3, of tumor tissue.
- Grade 2: Moderately effective
  - Viable cancer cells account for less than 1/3 of tumor tissue, while other cancer cells are severely degenerated or necrotic.
- Grade 3: Markedly effective
  - No viable cancer cells are evident.

- Note: Definite re-proliferation of tumor cells in treated cancer lesions, after preoperative treatment, should be recorded as “re-proliferation (+)”.

4.3. Lymph node metastasis (pN)

- Note 1: Lymph nodes should be sectioned through the hilum.
- Note 2: The number of dissected lymph nodes and metastatic lymph nodes should be recorded.
- Note 3: The metastatic ratio (the number of metastatic lymph nodes/the number of dissected lymph nodes) is described for each lymph node station. The total metastatic ratio is also described in parentheses.
  e.g.: No.104R (0/10), No.104 L (1/13), No.106recR (1/3), No.106recL (0/4).
- Note 4: Metastasis to soft tissue without a lymph node structure is described as extra-lymph node metastasis, and the locations and number of metastases are recorded.
  e.g.: lymph node metastasis, 1/25; extra-lymph node metastasis, 2/2.
- Note 5: Extranodal invasion including direct invasion and lymphatic invasion is described.
- Note 6: A lymph node with no viable cancer cells after preoperative treatment is diagnosed as negative for metastasis (pN0).

**Fig. 1-17:**
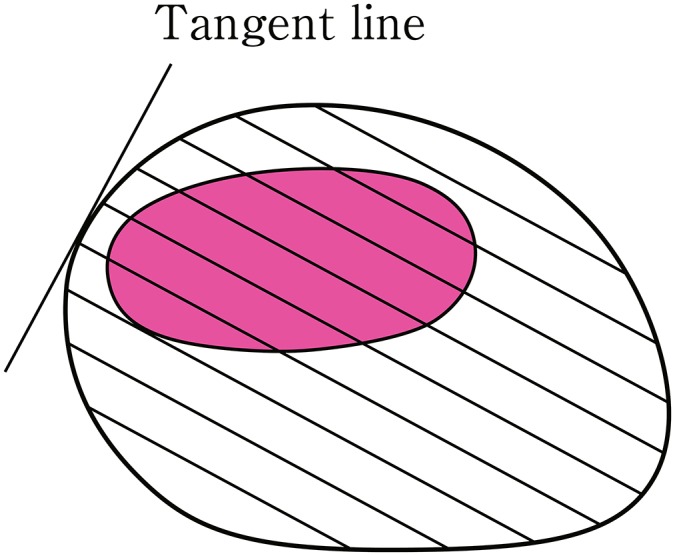
How to cut endoscopically resected specimens

[Reference]

Japanese Gastric Cancer Association: Japanese Classification of Gastric Carcinoma (in Japanese). 13th ed. Kanehara Shuppan, Tokyo, 1999; 27.

4.4. Distant organ metastasis (pM) (cf. 3.5 Distant organ metastasis (M))

4.5. Residual tumor (pR) (cf. 3.6 Residual tumor (R))

4.6. Curativity (pCur) (cf. Curativity (Cur))

**5. Endoscopic treatment**

5.1. Handling of specimens resected endoscopically^Note^

Extending fixation of the resected specimen: A specimen is extended and fixed immediately after resection on a cork board or polystyrene foam and is fixed in formalin solution of sufficient volume for at least half a day.

- Note: As for the extended fixation of the resected specimen, it should be done by the doctor or co-worker who carried out endoscopic treatment. Especially in piecemeal resection, fixation of the specimen should be performed by the doctor(s) aware of the actual figure of the tumor in vivo to enable more exact restructuring.

5.2. Description of macroscopic findings and endoscopic findings

An e-mark is prefixed to macroscopic findings and endoscopic findings.

*5.2.1 Number of tumors and number of resected specimens*

Number of lesions

Number of specimens resected from each tumor (number of specimens):

1. en bloc resection, 2. piecemeal resection.

*5.2.2 Size of resected specimen and size of tumor lesion (for each lesion)*

The size is described by the greatest longitudinal dimension in millimeters multiplied by the greatest transverse dimension in millimeters: a × b (mm).

*5.2.3 Tumor types*

The tumor types are classified into Type 0-I, Type 0-IIa, Type 0-IIb, Type 0-IIc, Type 0-III, combined type, and others.

*5.2.4 Macroscopic findings*

5.2.4.1. Horizontal margin (eHM)

eHMX: Whether residual tumor is present on the horizontal margin cannot be assessed.
eHM0: Non-cancerous squamous epithelium and lamina propria mucosae have been confirmed on all horizontal resection margins.
eHM1: The tumor is exposed on one of its horizontal resection margins.

5.2.4.2. Vertical margin (eVM)

eVMX: Whether residual tumor is present on the vertical margin cannot be assessed.
eVM0: The tumor is not exposed on any of its vertical margins.
eVM1: The tumor is exposed on one of its vertical margins.

*5.2.5. Clinical assessment of residual tumor* ^*Note 1, 2*^

eRX (non-assessable): Whether residual tumor is present on the resection margin cannot be assessed.
eR0 (complete resection): Non-cancerous squamous epithelium and lamina propria mucosa have been confirmed on all resection margins.
eR1 (slightly incomplete resection): Presence of an iodine-unstained area on the margin of the resected specimen.
eR2 (incomplete resection): Presence of residual tumor.

- Note 1: The clinical assessment of the residual tumor, referring to the iodine staining of the resected specimen, should be performed immediately after the endoscopic resection. In cases with a piecemeal resection, iodine staining of the ulcer margin after resection should be referenced.
- Note 2: This assessment method should be applied to squamous cell carcinoma.

5.3. Preparation for pathological examination (Fig. 1-17)

**Fig. 1-18:**
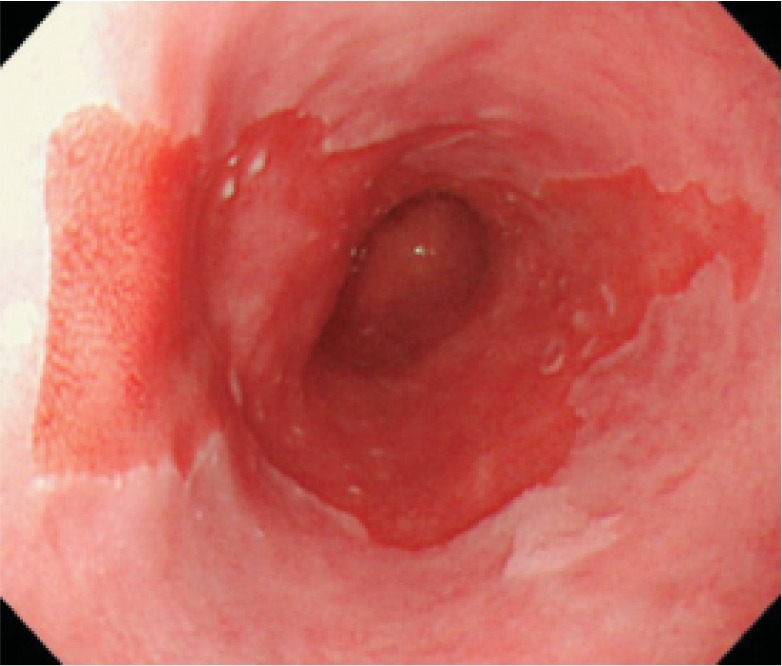
Long segment Barrett’s esophagus (LSBE)

Before cutting, formalin-fixed specimen should be stained with iodine solution to confirm unstained area.^Note^ Cutting lines are decided as crossing lines at right angles to the tangent line at the resection margin closest to the tumor, and a whole resected specimen is cut in slice each 2–3 mm thick.

- Note: Rinsing the sample with tap water for at least 30 min can result in a good staining condition. To make a clearer contrast between stained and unstained areas, the sample should be treated with relatively low concentration (0.1–0.5%) of iodine solution for a little bit longer time.

5.4. Description of pathological findings

The pathological diagnosis of an endoscopically resected specimen is summarized by the histological type, depth of tumor invasion, assessment of resection margin (horizontal and vertical), and vascular invasion.

*5.4.1. Pathological diagnosis*

The diagnosis is based on the histological classification (4.2.1). Although most esophageal tumors are squamous cell carcinoma, the evaluation of histological differentiation is omitted for intraepithelial carcinoma.

*5.4.2. Depth of tumor invasion (pT)*

A mucosal cancer is categorized in three depths of pT1a-EP, pT1a-LPM and pT1a-MM. In a submucosal cancer, the distance from the lamina muscularis mucosae is described because the entire submucosal layer cannot be examined in an endoscopically resected specimen. A submucosal cancer is sub-classified as pT1b-SM1 (submucosal tumor invasion limited to within 200 μm) and pT1b-SM2 (invasion to more than 200 μm).

e.g.: pT1b-SM2, 300 μm.

*5.4.3. Resection margin* ^*Note 1, 2*^

5.4.3.1. Horizontal margin (pHM)

pHMX: It cannot be assessed whether there is residual tumor on the horizontal margin or not.
pHM0: Non-cancerous squamous epithelium and lamina propria mucosae are confirmed on all horizontal resection margins.
pHM1: The tumor is exposed on any horizontal resection margin.

5.4.3.2. Vertical margin (pVM)

pVMX: It cannot be assessed whether there is residual tumor on the vertical margin or not.
pVM0: No tumor is exposed on any vertical margin.
pVM1: The tumor is exposed on any vertical margin.

- Note 1: When no tumor is recognized in any resection margin, it is defined as a complete resection (pR0), and when a tumor is recognized in any resection margin, it is defined as an incomplete resection (pR1).
- Note 2: When vascular invasion is present in the resection margin, it is defined as a positive resection margin (pHM1, pVM1).

5.4.3.3. Non-assessable resection margin (pRX)

1. Because of crushing injury or the burn effect in the specimen during endoscopic resection, non-cancerous tissue in the resection margin cannot be confirmed.

2. Reconstruction after piecemeal resection is impossible.^Note^

3. Suspected residual tumor in the basal layer because of non-continuous tumor extension.

4. Possible residual tumor in the vertical margin because of intra-ductal spread.

5. Indeterminable residual tumor because of other reasons.

- Note: In piecemeal resection pR0 is confirmed only when restructuring is possible and only non-cancerous tissue is recognized at the resection margins of the restructured specimen.

*5.4.4. Infiltrative growth pattern (INF)*

INFa (expansive type): Expansive growth of tumor nests with a well-demarcated border from the surrounding tissue.
INFb (intermediate type): Intermediate growth pattern, between INFa and INFc.
INFc (infiltrative type): Infiltrative growth of tumor nests with an ill-defined border from the surrounding tissue.

*5.4.5. Vascular invasion (ly/v)* ^*Note*^

It is not necessary to evaluate the degree of vascular involvement: only its presence or absence should be described.

5.4.5.1. Lymphatic invasion (ly)

ly (−): No lymphatic invasion.
ly (+): Lymphatic invasion

5.4.5.2. Venous invasion (v)

v (−): No venous invasion
v (+): Venous invasion

- Note: A special staining method for elastic fibers of the vascular wall, such as Elastica van Gieson (EVG) or Victoria blue (VB) staining, is needed to determine venous invasion. Immunostaining with an anti-D2-40 antibody is useful to confirm lymphatic invasion. When differentiating between lymphatic and venous invasion is difficult, the case should be described as ly/v. When lymphatic and/or venous invasion is prominent, this evaluation should be included in addition to ly (+) or v (+).

*5.4.6. Report of pathological findings*

All the above-mentioned factors should be described, and the attachment of a figure showing the general view of the resected specimen with the regional depths of tumor invasion and vascular invasion is recommended. It is better to attach a schematic figure showing pathological findings on the cut surface if necessary.

5.5 Residual tumor (pR)^Note 1,2^

pRX: The existence of residual tumor at the resection margin cannot be assessed pathologically.
pR0: No cancer tissue is pathologically present at any margin of the resected specimen.
pR1: Cancer tissue is pathologically present at the margin of the resected specimen.
pR2: A cancer lesion is present.

- Note 1: For a piecemeal resection, the presence of residual tumor is evaluated after rebuilding the specimen.
- Note 2: When multiple lesions are resected, each lesion is evaluated individually.

5.6. Curativity (pCur)

When endoscopic resection (EMR: endoscopic mucosal resection, or ESD: endoscopic submucosal dissection) is performed for superficial esophageal cancer that does not exhibit clinical metastasis, a comprehensive evaluation can be established, based on the pathological findings of depth of invasion, residual tumor, and vascular invasion (Table 1–9).

Curativity A (pCur A): pT1a-EP or pT1a-LPM with pR0.
Curativity B (pCur B): pT1a-EP or pT1a-LPM with pRX.
  pT1a-MM or pT1b-SM1 with pR0 or pRX.
Curativity C (pCur C): pT1b-SM2, positive micro vascular permeation despite depth of invasion, pR1 or pR2.

**Table 1-9:** Curativity of the endoscopic resection

Depth of tumor invasion (T)	Residual tumor (R)		Others
	pR0	pRX	pR1, pR2 and/or v + , ly+
pT1a-EP	A	B	C
pT1a-LPM	A	B	C
pT1a-MM	B	B	C
pT1b-SM1	B	B	C
pT1b-SM2	C	C	C

**6. Barrett esophagus and adenocarcinoma in Barrett esophagus**

6.1. Definition and description methods for Barrett mucosa, Barrett esophagus and Adenocarcinoma in Barrett esophagus

*6.1.1. Definition of the esophagogastric junction (EGJ)*

The EGJ should be defined systematically in accordance with the criteria listed below. Among these criteria, endoscopic findings should be given priority over findings obtained using other diagnostic modalities.

1. Endoscopic findings
  Lower margin of palisading small vessels
  If the palisading small vessels are unclear, the oral margin of the longitudinal folds of the greater curvature of the stomach is defined as the EGJ.
2. Upper gastrointestinal series (UGI)
  Narrowest locus of the lower esophagus
  In the presence of a sliding hiatal hernia, the upper end of the longitudinal folds is defined as the EGJ.
  In the presence of Barrett esophagus, the upper end of the longitudinal folds is defined as the EGJ.
3. Pathological study
  Macroscopic definition: The EGJ should be defined macroscopically as the point at which the luminal caliber changes in the area where the tubular esophagus is connected to the vestibule lumen of the stomach.
  Microscopic definition: For a mucosal layer with intact structures, the EGJ should be defined as follows:
  1. Non-Barrett esophagus: The EGJ is defined as the squamocolumnar junction.
  2. Barrett esophagus: Histological structures such as proper esophageal glands and their ducts, a double-layer muscularis mucosae, or palisading small vessels should be included in the microscopic definition of the EGJ.
  For a non-intact mucosal layer, the EGJ should be defined based on the macroscopic findings of the surgical specimen, and the EGJ should be presumed based on the presence of histological structures associated with the esophagus or stomach.

*6.1.2. Barrett mucosa*

Columnar epithelium continuous from the stomach with or without intestinal metaplasia

*6.1.3. Barrett esophagus*

An esophagus containing Barrett mucosa should be designated as Barrett esophagus.^Note 1^

At least one of the following conditions must be satisfied.

1. Presence of esophageal gland ducts in the mucosal layer or proper esophageal glands in the submucosal layer within the area of columnar epithelium.
2. Presence of squamous islands in the columnar epithelium.
3. Presence of a double-layer muscularis mucosae^Notes 2,3^

- Note 1: The presence of circular Barrett mucosa extending longitudinally for 3 cm or more is called long segment Barrett esophagus (LSBE) (Fig. 1-18). On the other hand, the presence of circular Barrett mucosa less than 3 cm in length or the presence of non-circular Barrett mucosa is designated as short segment Barrett esophagus (SSBE) (Fig. 1-19).

**Fig. 1-19:**
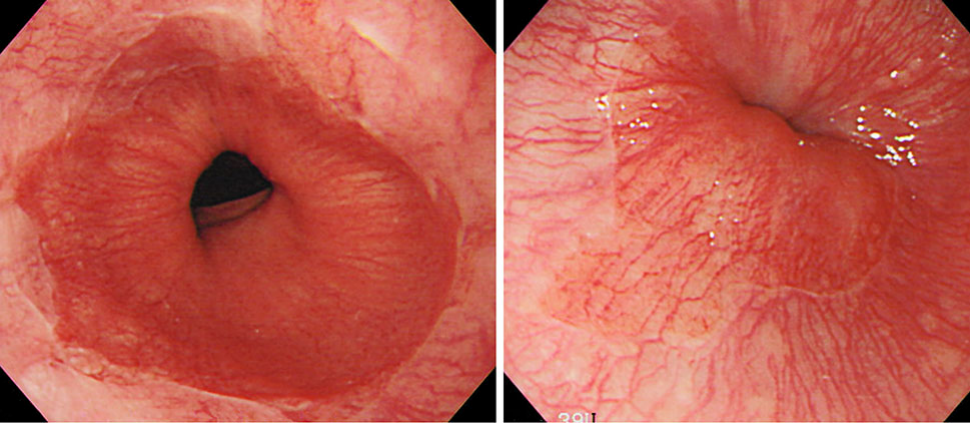
Short segment Barrett esophagus (SSBE). Circular Barrett mucosa extending for less than 3 cm in length or non-circular Barrett mucosa is designated as short segment Barrett esophagus (SSBE). **a** Circular SSBE. **b** Non-circular SSBE
- Note 2: New muscularis mucosae can sometimes be found immediately under the columnar epithelium. In the Japanese Classification, the primary muscularis mucosae is called deep muscularis mucosae (DMM), and the new muscularis mucosae is called superficial muscularis mucosae (SMM). The identification of SMM and DMM is occasionally difficult because of the fusion of both layers, the thickness of the layer, or the presence of irregularities.
- Note 3: Barrett esophagus can also be defined as the presence of columnar epithelium containing small palisading vessels with diameters of greater than 100 µm within the lamina propria mucosae.

*6.1.4. Adenocarcinoma in Barrett esophagus*

Adenocarcinoma arising in Barrett mucosa^Note 1^

- Note 1: The origin of adenocarcinoma in areas of Barrett esophagus is not the same as that of gastric adenocarcinoma, which is derived from gastric mucosa adjacent to the EGJ. Gastric cancer is described according to the Japanese Classification of Gastric Cancer, and esophageal adenocarcinoma is described according to the Japanese Classification of Esophageal Cancer. However, the histological classifications of both adenocarcinomas are described according to the Japanese Classification of Gastric Cancer.

Circular Barrett mucosa extending longitudinally for 3 cm or more is called long segment Barrett esophagus (LSBE).

6.2. Tumor location

Same as that for esophageal cancer.

- Note: In cases with a hiatus hernia, the tumor location should be determined using barium contrast radiography.

6.3. Description of tumors

Adenocarcinoma in Barrett esophagus is described according to the Japanese Classification for Esophageal Cancer, except for the depth of tumor invasion.

*6.3.1. Primary tumor*

6.3.1.1. Circumferential location

6.3.1.2. Tumor size

6.3.1.3. Macroscopic tumor types

6.3.1.4. Depth of tumor invasion (T)

TX Depth of tumor invasion cannot be assessed.
T0 No evidence of primary tumor.
T1a Tumor has invaded the mucosa.
  1. Tumor has invaded the superficial muscularis mucosae (SMM).
  2. Tumor has invaded the lamina propria mucosae.
  3. Tumor has invaded the deep muscularis mucosae (DMM).
T1b Tumor has invaded the submucosa (SM).
  1. Tumor has invaded the upper third of the submucosa.
  2. Tumor has invaded the middle third of the submucosa.
  3. Tumor has invaded the lower third of the submucosa.
T2 Tumor has invaded the muscular propria.
T3 Tumor has invaded the adventitia.
T4 Tumor has invaded adjacent structure(s).

*6.3.2. Intramural metastasis (IM)*

Same as that for esophageal cancer.

*6.3.3. Lymph node metastasis (N)* ^*Note*^

- Note: In cases with a hiatal hernia, the field of lymph node dissection should be designated according to the tumor location as defined using Barium contrast radiography.

*6.3.4. Distant organ metastasis (M)*

Same as that for esophageal cancer.

6.4. Stage

Same as that for esophageal cancer.

**7. Treatment**

7.1. Endoscopic treatment

*7.1.1. Endoscopic resection: ER*

7.1.1.1. Endoscopic mucosal resection: EMR

7.1.1.2. Endoscopic submucosal dissection: ESD

- Note: The following items should be described.

Method of resection: (1) en bloc resection, (2) piecemeal resection.

Residual tumor: eRX, eR0, eR1, eR2.

Complications: (1) perforation, (2) bleeding requiring treatment, (3) stenosis, (4) others (including mediastinal emphysema).

Combined therapy: (1) none, (2) APC, (3) laser, (4) PDT, (5) MCT, (6) others.

*7.1.2. Other Endoscopic treatments*

7.1.2.1. Argon plasma coagulation: APC

7.1.2.2. Laser therapy: laser

7.1.2.3. Photodynamic therapy: PDT

7.1.2.4. Microwave coagulation therapy: MCT

7.1.2.5. Others

- Note: Any other therapy performed should be described.

7.2. Surgical treatments

*7.2.1. Resection and reconstruction procedures*

7.2.1.1. Staged operations

One-stage operation

Staged operation

7.2.1.2. Surgery with multi-modality treatments

Planned surgery: planned surgery after neoadjuvant chemotherapy, radiotherapy, or both.

Salvage surgery: surgery for cases with residual tumor or cases with recurrent tumor after definitive chemoradiotherapy with more than 50 Gy radiation.

The surgical methods, such as esophagectomy, lymphadenectomy, endoscopic resection, and so on, should be described.

7.2.1.3. Approaches for tumor resection

- Endoscopic
- Thoracoscopic, thoracoscopy-assisted
- Mediastinoscopic
  - Laparoscopic, laparoscopy-assisted
  - Transcervical
  - Thoracotomy
    - Right
    - Left
  - Laparotomy
  - Thoracoabdominal incision
    - Right
    - Left
  - Transhiatal
  - Sternotomy

- Note: When several approaches are adopted, only the main approach should be described.

7.2.1.4. Extent of esophageal resection

Total esophagectomy: the cervical, thoracic and abdominal esophagus is resected, regardless of whether laryngectomy is done or not.

Subtotal esophagectomy: almost all the thoracic esophagus is resected

Middle and lower esophagectomy: the middle and lower esophagus (including abdominal esophagus) is resected ^Note 1^

Lower esophagectomy: the lower esophagus (including abdominal esophagus) is resected

Partial esophagectomy: resection of full-thickness partial esophagus^Note 2^

Mucosal resection: resection of mucosal and submucosal layers

Others

- Note 1: Lower esophagectomy includes resection of the lower esophagus and cardia.
- Note 2: In partial esophagectomy, the location of the esophagus resected should be described.

7.2.1.5. Combined resection

The organ(s) resected together because of cancer invasion should be described.

- Note: Total or partial resection of the organ(s) should be described.

7.2.1.6. Reconstruction

7.2.1.6.1. Reconstruction routes

Antethoracic (subcutaneous)

Retrosternal

Posterior mediastinal

7.2.1.6.2. Sites of anastomosis

Neck

Antethoracic (subcutaneous)

Thoracic cavity (proximal, distal)

Lower mediastinum

- Note: The border between the proximal and the distal site of anastomosis is the upper level of the aortic arch.

7.2.1.6.3. Organs used for reconstruction

- Stomach
  - Whole stomach
  - Gastric tube
- Jejunum
  - Pedicled
  - Free
- Colon
  - Pedicled^Note 1^
    - Left colon^Note 2^
    - Right colon^Note 3^
    - Ileum and colon
- Free
  - Colon
  - Ileum and colon
- Skin and muscle^Note 4^
  - Skin flap
    - Local skin flap
    - Pedicled
    - Free
  - Musculocutaneous flap
    - Pedicled
    - Free

- Note 1: Isoperistaltic or antiperistaltic should be described.
- Note 2: For reconstruction with left colon, the transverse colon with left colic artery is used.
- Note 3: For reconstruction with right colon, the ascending colon with the middle colic artery is used.
- Note 4: The name of the skin flap or muscle flap that is used should be described.
  e.g.: free forearm skin flap, right pectoralis major musculocutaneous flap.

*7.2.2. Conservative/palliative procedures*

7.2.2.1. Stoma

Pharyngostomy

Esophagostomy

Gastrostomy

Jejunostomy

7.2.2.2. Bypass

Pharyngogastrostomy

Esophagogastrostomy

Esophagojejunostomy

Esophagocolostomy

7.2.2.3. Exploratory thoracotomy, exploratory laparotomy

7.2.2.4. Others

Lymph node dissection without esophagectomy

7.3. Stenting

*7.3.1. Esophageal stents*

- Therapy before stenting
  No
  Yes (chemotherapy, radiotherapy, chemoradiotherapy, others)
- Therapy after stenting
  No
  Yes (chemotherapy, radiotherapy, chemoradiotherapy, others)
- Esophageal fistula
  No
  Yes
- Type of stent
  Covered or non-covered
  Anti-reflux valve (yes, no)
  Diameter
  Length
- Operative complications
  No
  Yes (bleeding requiring treatment, dyspnea, pain, others)
- Evaluation of stent
  Status of eating before stenting
  Status of eating at discharge
- Complications after stenting
  No
  Yes (bleeding requiring treatment, perforation, pain, regurgitation of gastric contents, dyspnea, migration, esophago-airway fistula, others)

*7.3.2. Tracheobronchial stents*

Type of stent.

*7.3.3. Aortic stents*

Type of stent.

7.4. Common issues for radiotherapy and chemotherapy

*7.4.1. Disease status*

Untreated

Macroscopic residual after surgery

Microscopically residual after surgery

No obvious residual disease after surgery

Loco-regional recurrence after surgery

Distant metastasis after surgery

Other postoperative recurrence

Residual disease after EMR/ESD

No obvious residual disease after EMR/ESD

After stenting

After intraoperative radiation therapy for macroscopic disease

After intraoperative radiation therapy for no obvious macroscopic disease

*7.4.2. Aim of treatment*

Definitive

Palliative

Preoperative

Preventive

Recurrent disease

Others

*7.4.3. Reasons for definitive radiotherapy*

Severe associated disease(s)

Advanced age

Patient’s wishes

Others

7.5. Radiotherapy (RT)

*7.5.1. Clinical target volume (CTV)*

Primary lesion

Entire esophagus

Resected lymph node area for prevention (supra-clavicular, mediastinal, abdominal)

Distant organ metastasis

*7.5.2. Methods of radiotherapy*

External beam radiation therapy

External beam radiation therapy + intra-cavitary radiation therapy

Intra-cavitary radiation therapy alone

*7.5.3. External beam radiotherapy*

X-ray

Proton

Carbon

Gamma-ray

Electron

7.5.3.1. Planning methods

X-ray simulator

Three dimensional

7.5.3.2. Field setting

Opposing

Anterior oblique

Three directions

More than 4 fields

Rotating

Intensity-modulated radiotherapy (IMRT)

7.5.3.3. Reference points

CTV center

Field center

Others

7.5.3.4. Dose calculation

Non-homogeneity correction

Algorithm

7.5.3.5. Dose fractionation of external beam radiotherapy

Dose/fraction

Number of fractions/week

Total dose

Overall treatment time

*7.5.4. Intraluminal irradiation*

Low dose rate (^226^Ra)

High dose rate (^192^Ir, ^60^Co, ^137^Cs)

7.5.4.1. Reference points

Mucosal surface

____mm below mucosa

7.5.4.2. Dose fractionation of intraluminal irradiation

Dose/fraction

Number of fractions/week

Total dose

Overall treatment time

7.5.5. Completion of treatment

Complete without break

Complete with break

Incomplete

7.5.6. Reasons for treatment cessation

Completion of the planned treatment

Disease progression

Adverse events

Patient’s refusal (related to adverse events)

Patient’s refusal (not related to adverse events)

7.6. Chemotherapy (CT)

*7.6.1. Agents*

Name of agent (generic name should be recorded).

*7.6.2. Administration routes*

Intravenous

Oral

Transarterial

Local injection (including abdominal and chest cavity)

Others

*7.6.3. Administration procedures*

Bolus

Continuous

Others

*7.6.4. Administration doses*

Dose should be recorded as per body surface area (/m^2^) or per body (/body).

*7.6.5. Administration schedules*

Course duration

Course interval

Upper limit in number of courses.

*7.6.6. Duration of administration*

Initial date of administration

Last date of administration

Total number of courses

*7.6.7. Total administration dose*

Total administration dose of each agent should be calculated as per body surface area or per body.

*7.6.8. Reasons for treatment cessation*

Completion of the planned treatment

Disease progression

Adverse events

Patient’s refusal (related to adverse events)

Patient’s refusal (not related to adverse events)

Others

*7.6.9. Adverse events*

Recorded in accordance with the “Common Terminology Criteria for Adverse Events ver. 4, (Japanese version) JCOG/JSCO edition”.

7.7. Multi-modality treatment

*7.7.1 Combination of endoscopic treatment and surgery, radiotherapy, chemoradiotherapy or chemotherapy*

Classification: Preoperative, intraoperative, or postoperative.
Description: Planned treatment or salvage treatment
Surgery: Evaluation of residual tumor (pR)
  (If a residual tumor is present, describe the tumor location, depth of tumor invasion, tumor margin, presence of vascular invasion, presence of lymph node metastasis, etc.)
  Multiple tumors: yes or no
  (If multiple tumors are present, describe the tumor location and the number of lesions.)
  - Note: Evaluate residual tumors of primary and metastatic lesions.

Radiotherapy: radiation field, total dose

Chemotherapy: regimen

*7.7.2 Chemoradiotherapy (CRT)*

Classification according to timing: concurrent or sequential

Classification according to intent: definitive, neoadjuvant, or after surgery (adjuvant, additive)

- Note: R0 resection should be recorded as adjuvant. R2 should be recorded as additive.

7.8. Hyperthermia (HT)

7.9. Immunotherapy (IT)

**8. Results of treatment**

The following matters are recorded to allow precise statistical analysis for a comprehensive registry of esophageal cancer.

8.1. Total number of patients

Total number of outpatients

Total number of admitted patients

Total number of patients admitted for various treatments

8.2. Multiple primary cancers

Primary lesion of another cancer, diagnosis (clinical and pathological), synchronous or metachronous, and treatment

8.3. Main treatment and adjuvant therapy

Endoscopic treatment

Surgical treatment

Palliative operation

Radiotherapy

Chemotherapy

Chemoradiotherapy

Other non-surgical treatment

No treatment

8.4. Total number of patients treated, and number and rate of patients treated with each procedure

*8.4.1. Patients operated*

The total number of patients admitted, the number of patients who underwent resection, the numbers of patients with curative and non-curative resections, and the resection rate should be recorded.

Resection rate = patients who underwent resection/patients admitted^Note 1^

Total number and ratio of patients with or without curative resection^Notes 2, 3^

- Note 1: Patients who have undergone an esophagectomy for the first time are evaluated.
- Note 2: Curative operation is defined as Curativity A or B resection.
- Note 3: Non-curative operation is defined as Curativity C resection.

*8.4.2. Patients with Endoscopic treatment*

Record the number of patients who underwent endoscopic treatment, and the total number and ratio of patients with or without curative resection.

Cases of treatment completed only by endoscopic treatment are recorded separately from operated cases as endoscopically treated cases.

One-piece resection rate = (number of patients who underwent one-piece resection/total number of patients who underwent endoscopic resection) × 100^Note^

Curativity rate = (number of patients who underwent curative endoscopic resection/total number of patients who underwent endoscopic resection) × 100

- Note: The number of patients who underwent endoscopic resection indicates those who underwent endoscopic mucosal dissection and endoscopic submucosal dissection. It does not include the number of patients treated by laser therapy or photodynamic therapy.

*8.4.3. Patients with chemotherapy and/or radiotherapy*

Total numbers and rates according to response evaluation criteria after treatment.

8.5. Operative mortality^Note^

Operative mortality = (operative deaths/patients operated) × 100

Mortality after esophagectomy = (deaths after esophagectomy/patients who underwent esophagectomy) × 100

- Note: Operative death means death within 30 days after operation in or out of hospital.

8.6. Hospital mortality^Notes 1, 2^

Hospital mortality = (hospital deaths/patients operated) × 100

Hospital mortality after esophagectomy = (hospital deaths after esophagectomy/patients who underwent esophagectomy) × 100

- Note: Hospital death is defined as death during the same hospitalization, regardless of department at time of death.

8.7. Long-term outcome

The following items should be recorded for survival analysis.

*8.7.1. Alive or dead*

Alive: The date of the most recent follow-up

Death: The date of death

Unknown: The date of the most recent follow-up

- Cause of death
  - Treatment-related death: death because of surgical treatment, chemotherapy, radiotherapy, or other therapies
  - Death because of esophageal cancer
  - Death because of another cancer: primary cancer-related deaths should be recorded.
  - Death because of another disease: the name(s) of the disease(s) should be recorded.
  - Death because of accident: suicide should be included.
  - Death because of unknown cause(s): this category should be basically regarded as esophageal cancer-related deaths.

8.7.2. Recurrence

- Yes or No
- Date of recurrence
- Pattern and site of recurrence: Each recurrence should be recorded chronologically
- Local recurrence
  - Primary lesion (esophagus)^Note 1^
  - Recurrence in the mediastinum adjacent to the primary lesion
  - Recurrence at the anastomotic site or in the esophageal stump
  - Recurrence in the regional lymph nodes
  - Others including intramural metastasis in the esophagus or stomach
- Distant metastasis^Note 2^
  - Lymphogenous recurrence
  - Hematogenous recurrence (distant organ(s))
  - Disseminated recurrence (pleura, peritoneum, pericardium)
- Unknown

- Note 1: Recurrence at the same place as the primary lesion in the esophagus can occur after esophagus-preserving treatment including endoscopic mucosal resection, chemotherapy and radiotherapy.
- Note 2: Recurrent organ(s) are indicated by the abbreviations of the TNM classification.

Liver: HEP, Lung: PUL, Peritoneum: PER, Lymph node: LYM, Bone: OSS.

Brain: BRA, Kidney: REN, Adrenal gland: ADR, Skin: SKI, Others: OTH.

8.8. Long-term outcomes and prognosis, especially survival rate

*8.8.1. Analysis of survival rates*

Target (operation, endoscopic treatment, curativity and so on)

The calculation method of survival rates

Crude survival rate: direct method, cumulative method (Life-table method, Kaplan–Meier method)

Relative survival rate

Censored cases: overall survival rate, cause-specific survival rate

Statistical analysis of survival rate

Rate of cases lost to follow-up

*8.8.2. Period and rate of esophageal preservation*

This is the period during which the esophagus is preserved in patients with esophageal cancer who underwent non-esophagectomy treatment such as endoscopic mucosal resection, endoscopic submucosal dissection, chemotherapy and/or radiotherapy. The rate of those patients per all patients who underwent treatment for esophageal cancer is described.

[Reference]

Murakami M, Kuroda Y, Matsumoto S, et al. Treatment results of esophageal carcinoma of clinical T3, T4 M0: histological comparison between neoadjuvant chemoradiotherapy followed by surgery or definitive radiotherapy and conventional surgery. Oncol Rep 2000; 7: 571–578.

8.9. Terminology related to survival period

*8.9.1. Survival time*

Time until death from the time of a certain event

*8.9.2. Overall survival (OS)*

Time until death regardless of cause from the initial date. In observation studies, the day starting treatment, or the day confirming diagnosis is used as the initial date. In clinical trials, the “registration date” (the allocation day in the case of a randomized controlled trial) is used.

*8.9.3. Median survival time (MST)*

Period from the initial date to the first date of the survival rate being less than 50% in the survival curve calculated by the Kaplan–Meier method. The initial date is the same as described in 8.9.2.

*8.9.4. Survival rate*

The rate of survivors at a given time

*8.9.5. Progression-free survival (PFS), time to progression (TTP)*

It is the shortest period among periods from the initial date to progression or recurrence, or death. Death from any cause is regarded as an event, in PFS. Death is only regarded as an event if it is caused by esophageal cancer, in TTP.

*8.9.6. Relapse-free survival, recurrence-free survival (RFS)*

This is the period between the initial date to recurrence or death.

The day when the disease-free state was achieved such as an operation day, is used as the initial date.

*8.9.7. Disease-free survival (DFS)*

It is the shortest period among the periods from the initial date to a recurrence, death, or diagnosis of a second primary cancer. The day when the disease-free state such as an operation day was achieved, is used as the initial date.

*8.9.8. Time to treatment failure (TTF)*

This is the shortest period among the periods from the initial date to treatment cessation, progression, and death.

*8.9.9. Response duration*

Period from the first day when the disease was assessed as CR or PR until progression.

*8.9.10. Complete response duration*

Period from the first day when the disease was assessed as CR until recurrence.

[Reference]

Japan Society for Clinical Oncology. Terminology in Clinical Oncology 2013 (in Japanese)

